# Systemic Delivery of MicroRNA-101 Potently Inhibits Hepatocellular Carcinoma *In Vivo* by Repressing Multiple Targets

**DOI:** 10.1371/journal.pgen.1004873

**Published:** 2015-02-18

**Authors:** Fang Zheng, Yi-Ji Liao, Mu-Yan Cai, Tian-Hao Liu, Shu-Peng Chen, Pei-Hong Wu, Long Wu, Xiu-Wu Bian, Xin-Yuan Guan, Yi-Xin Zeng, Yun-Fei Yuan, Hsiang-Fu Kung, Dan Xie

**Affiliations:** 1 The State Key Laboratory of Oncology in South China, Sun Yat-Sen University Cancer Center, Collaborative Innovation Center for Cancer Medicine, Guangzhou, China; 2 Medical Research Center, Sun Yat-Sen Memorial Hospital, Guangzhou, China; 3 Department of Pathology, Sun Yat-Sen University Cancer Center, Guangzhou, China; 4 Tumor Interventional Therapy, Sun Yat-Sen University Cancer Center, Guangzhou, China; 5 Hepatobiliary Oncology, Sun Yat-Sen University Cancer Center, Guangzhou, China; 6 Department of Clinical Oncology, People’s Hospital, Wuhan University, Wuhan, China; 7 Institute of Pathology and Southwest Cancer Center, Southwest Hospital, Third Military Medical University, Chongqing, China; 8 Department of Clinical Oncology, the University of Hong Kong, Hong Kong, China; 9 State Key Laboratory of Oncology in South China, the Chinese University of Hong Kong, Hong Kong, China; St Jude Children’s Research Hospital, United States of America

## Abstract

Targeted therapy based on adjustment of microRNA (miRNA)s activity takes great promise due to the ability of these small RNAs to modulate cellular behavior. However, the efficacy of miR-101 replacement therapy to hepatocellular carcinoma (HCC) remains unclear. In the current study, we first observed that plasma levels of miR-101 were significantly lower in distant metastatic HCC patients than in HCCs without distant metastasis, and down-regulation of plasma miR-101 predicted a worse disease-free survival (DFS, P<0.05). In an animal model of HCC, we demonstrated that systemic delivery of lentivirus-mediated miR-101 abrogated HCC growth in the liver, intrahepatic metastasis and distant metastasis to the lung and to the mediastinum, resulting in a dramatic suppression of HCC development and metastasis in mice without toxicity and extending life expectancy. Furthermore, enforced overexpression of miR-101 in HCC cells not only decreased EZH2, COX2 and STMN1, but also directly down-regulated a novel target ROCK2, inhibited Rho/Rac GTPase activation, and blocked HCC cells epithelial-mesenchymal transition (EMT) and angiogenesis, inducing a strong abrogation of HCC tumorigenesis and aggressiveness both *in vitro* and *in vivo*. These results provide proof-of-concept support for systemic delivery of lentivirus-mediated miR-101 as a powerful anti-HCC therapeutic modality by repressing multiple molecular targets.

## Author Summary

Human hepatocellular carcinoma (HCC) is one of the most common malignancy worldwide and among the leading causes of cancer-related death. HCC is often diagnosed at an advanced stage and there is still no effective therapeutic strategy for non-resectable HCCs. It has been suggested that the therapeutic delivery of certain miRNA(s) has a unique advantage in clinical use. We first find that the plasma levels of miR-101 are significantly down-regulated in HCC patients with distant metastasis and associated closely with HCCs progression and/or worse disease-free survival (DFS). Next, we identify that systemic delivery of lentivirus-mediated miR-101 in an orthotopic liver implanted HCC model of mouse, not only suppresses tumor xenograft growth in the liver, but also substantially blocks intrahepatic metastasis and distant metastasis to the lung and to the mediastinum, resulting in a dramatic abrogation of HCC tumorigenesis and progression in mice without toxicity. Furthermore, functional and/or mechanistic studies of miR-101 demonstrate that miR-101 in HCC cells inhibits Rho/Rac GTPase activation, and blocks HCC cells epithelial-mesenchymal transition (EMT) and angiogenesis, inducing a strong abrogation of HCC tumorigenesis and aggressiveness both *in vitro* and *in vivo*.

## Introduction

Hepatocellular carcinoma (HCC) is one of the most common malignancy worldwide [1]. In China, HCC is the second highest cancer killer, which along accounts for 53% of all liver cancer deaths in the world [2]. HCC is often diagnosed at an advanced stage and there is no effective therapeutic strategy for non-resectable HCCs. so far, since highly active drug-metabolizing pathways and multidrug resistance transporter proteins in tumor cells always diminish the efficacy of current therapeutic regimens for HCC. Therefore, alternative modalities of treatment are urgently needed for this uniformly fatal disease.

MicroRNA (miRNA)s are a class of highly conserved short RNAs that suppress gene expression [3] and have a functional contribution to cellular transformation and/or tumorigenesis [4,5]. In human neoplasms, some miRNAs are often up-regulated and may perform an oncogenic function, while most miRNAs are down-regulated and have a tumor suppressive activity [6]. Thus, potential therapeutic approaches in diseases, such as cancer, that target specific miRNAs have recently attracted attention [7]. Inhibition of oncogenic miRNAs through the use of antisense reagents is clearly one of the approaches [8]. On the other hand, miRNA-replacement therapy is another efficacious strategy [9]. In HCC, it was reported that systemic administration of miRNA (miR)-26a in a transgenic mouse HCC model could result in a dramatic suppression of HCC cell proliferation, induction of tumor-specific apoptosis and protection from disease progression without toxicity [10].

Recently, we and other groups have found that the levels of a specific miRNA, miR-101, were frequently down-regulated in human HCC tissues, and ectopic overexpression of miR-101 dramatically inhibited HCC cells tumorigenicity and invasiveness *in vitro* by targeting *MCL-1* and *FOS*, respectively [11,12]. More recently, it has been reported that miR-101 could inhibit autophagy and enhance cisplatin-induced apoptosis in HCC cells by targeting *STMN1* [13]. In other solid tumors, the levels of miR-101 were also decreased in neoplastic tissues [14–17], and miR-101 could inhibit the tumorigenesis and/or cancer progression by repressing the oncogenes *EZH2 and COX2* [17–20]. These data suggest a powerful anti-tumorigenic activity of miR-101 in different human cancers. To date, however, the *in vivo* efficacy of miR-101 replacement therapy to human cancers, such as HCC, has not been elucidated. In the current study, we thus determined to investigate the therapeutic efficacy of systemic delivery of lentivirus-mediated miR-101 in an orthotopic liver implanted HCC model of mouse, and the tumor repressive functions of miR-101 in HCC and underling mechanisms were further studied.

## Results

### Down-regulation of plasma MiR-101 is a frequent event in HCC patients with distant metastasis and predicts worse prognosis

**Figure 1:**
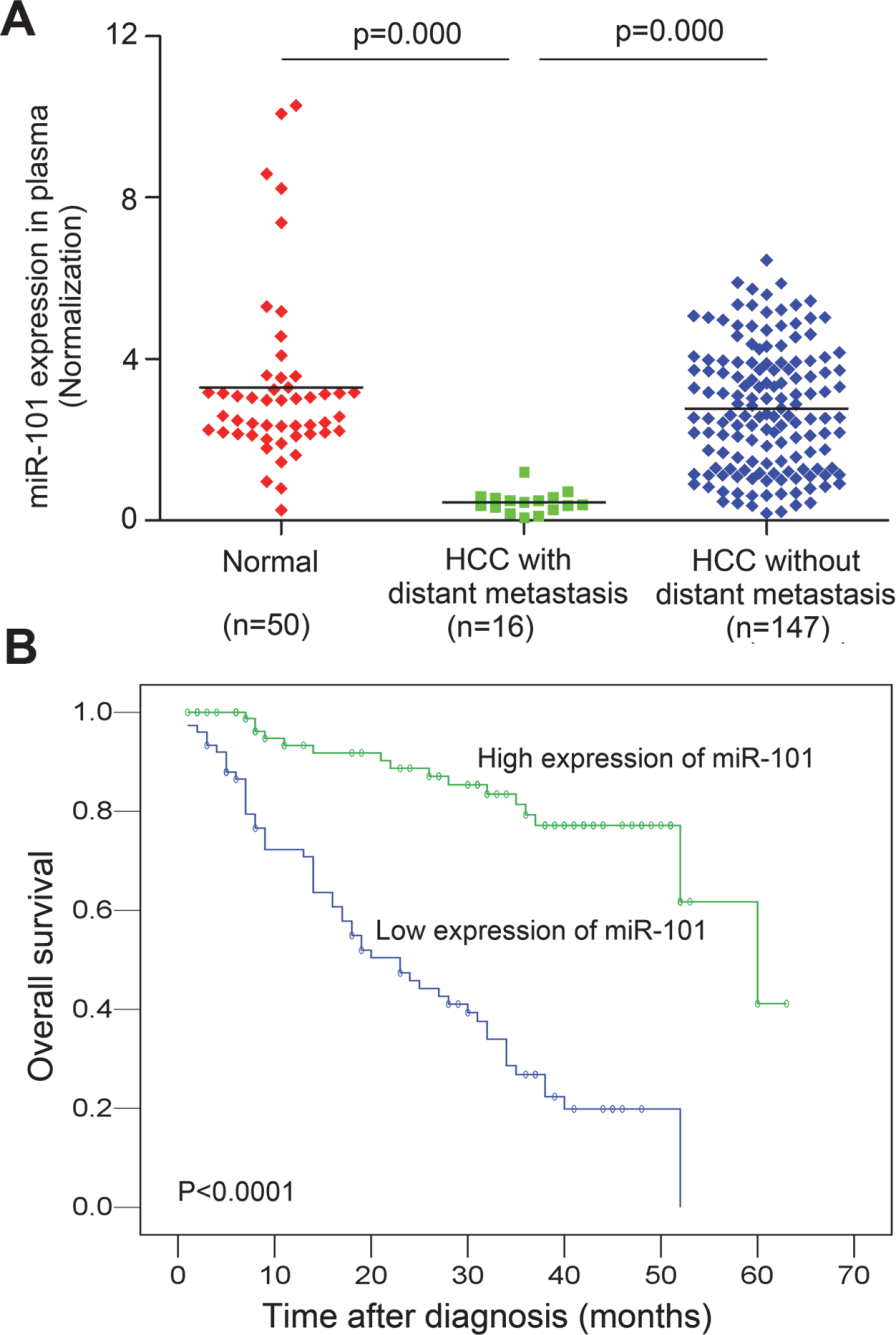
Analysis of miR-101 levels in human plasma samples by real-time PCR and Kaplan-Meier analysis for HCC patients DFS according to the plasma levels of miR-101. **A.** Expression levels of miR-101 in human plasma samples from healthy donors (*n* = 50) and HCC patients with distant metastasis (*n* = 16) and without distant metastasis (*n* = 147). **B.** Kaplan-Meier analysis for HCC patients DFS according to plasma levels of miR-101. The levels of miR-101 was analyzed by real-time PCR, and ROC curve analysis was applied to determine the cutoff score for high expression (*n* = 88) and low expression of plasma miR-101 (*n* = 75).

The plasma levels of miR-101 were examined by Real-time PCR in 163 HCC patients and 50 healthy donors. To identify a single, optimal cutpoint for mature miR-101, ROC curve analysis was applied to our HCC cohort to determine the cutoff score for high or low expression of miR-101 [21]. Tumors designated as “high expression” for miR-101 were those with scores above the value of 2.243928. The average plasma levels of miR-101 were significantly lower in HCC patients with distant metastasis than that in HCCs without distant metastasis and control healthy donors (Fig. 1A). High expression of plasma miR-101 was examined in 88/163 (54.0%) of HCC patients. Correlation analysis showed that low level of plasma miR-101 in HCC patients was significantly associated with a more aggressive phenotype (Table 1, p<0.05). Further survival analysis established that the plasma level of miR-101 is an independent prognostic factor for HCC patient survival (p<0.0001, Fig. 1B, Table 2).

**Table 1:** Correlation of plasma miR-101 expression with patients’ clinicopathologic variables in human hepatocellular carcinomas.

	**miR-101**			
**Variable**	**All cases**	**Low** **expression**	**High expression**	***P* value** *
Age (years)				0.231
≤48.2^†^	80	33 (41.3%)	47 (58.8%)	
> 48.2	83	42 (50.6%)	41 (49.4%)	
Sex				0.306
Male	136	65 (47.8%)	71 (52.2%)	
Female	27	10 (37.0%)	17 (63.0%)	
Etiology				0.581
HBV	134	63 (47.0%)	71 (53.0%)	
None	29	12 (41.4%)	17 (58.6%)	
AFP (ng/ml)				0.405
≤20	84	36 (42.9%)	48 (57.1%)	
>20	79	39 (49.4%)	40 (50.6%)	
Liver cirrhosis				0.272
Yes	108	53 (49.1%)	55 (50.9%)	
No	55	22 (40.0%)	33 (60.0%)	
Tumor size (cm)				0.001
≤5	85	29 (34.1%)	56 (65.9%)	
>5	78	46 (59.0%)	32 (41.0%)	
Tumor multiplicity				0.000
Single	111	40 (36.0%)	71 (64.0%)	
Multiple	52	35 (67.3%)	17 (32.7%)	
Differentiation				0.172
Well	22	7 (31.8%)	15 (68.2%)	
Moderate	93	48 (51.6%)	45 (48.4%)	
Poor	40	15 (37.5%)	25 (62.5%)	
Undifferentiated	8	5 (62.5%)	3 (37.5%)	
Stage				0.000
I	20	3 (15.0%)	17 (85.0%)	
II	61	15 (24.6%)	46 (75.4%)	
III	53	30 (56.6%)	23 (43.4%)	
IV	29	27 (93.1%)	2 (6.9%)	
Distant metastasis				
M1	16	16 (100%)	0 (0%)	
MX	147	61 (40.8%)	86 (59.2%)	

*Chi-square test

^†^Mean age

HBV indicates hepatitis B virus

AFP indicates alpha-fetoprotein.

**Table 2:** Univariate and multivariate analysis of different prognostic factors in 163 patients with hepatocellular carcinoma.

	**Univariate analysis** *			**Multivariate analysis** **^†^**	
**Variable**	**All cases**	**Mean survival (months)**	***P* value**	**HR (95% CI)**	***P* value**
Age (years)			0.491		
≤48.2^‡^	80	39.4			
> 48.2	83	37.0			
Sex			0.349		
Male	136	37.9			
Female	27	39.1			
Etiology			0.767		
HBV	134	33.5			
None	29	38.7			
AFP (ng/ml)			0.000	2.111 (1.219–3.654)	0.008
≤20	84	47.3			
>20	79	28.6			
Liver cirrhosis			0.766		
Yes	108	37.7			
No	55	39.1			
Tumor size (cm)			0.001	1.348 (0.784–2.318)	0.281
≤5	85	45.9			
>5	78	31.3			
Tumor multiplicity			0.000	1.462 (0.827–2.584)	0.191
Single	111	45.7			
Multiple	52	24.3			
Differentiation			0.273		
Well/moderate	115	39.8			
Poor/undifferentiated	48	34.7			
Stage			0.000	2.733 (1.375–5.434)	0.004
I/II	81	50.2			
III/ IV	82	27.0			
Distant metastasis			0.000	1.541 (0.811–2.929)	0.187
MX	147	41.0			
M1	16	18.2			
miR-101 expression			0.000	0.480 (0.270–0.852)	0.012
Low expression	75	25.2			
High expression	88	51.5			

*Log-rank test

^†^Cox regression model

^‡^mean age

HR indicates hazards ratio

CI indicates confidence interval

HBV indicates hepatitis B virus

AFP indicates alpha-fetoprotein.

It has been reported that HBx-mediated miR-101 down-regulation and subsequent induction of aberrant DNMT3A expression contributes to HBV mediated hepatocarcinogenesis [22]. We thus examined the levels of miR-101 in HBV-negative and HBV-positive HCC patient’s plasma. We found that there are no significant differences between the plasma levels of miR-101 in HBV-negative and HBV-positive HCC patients (S1A Fig.). At the same time, so are the results in HBV-negative and HBV-positive HCC patient’s plasma with distant metastasis (S1B Fig.). Consequently, it is unlikely that HBV infection itself induced the differential expression patterns of plasma miR-101 in our set of HCCs.

### Therapeutic delivery of miR-101 suppresses tumor growth, angiogenesis and metastasis in an orthotopic liver implanted HCC model of mouse

In our study, we subsequently assessed the therapeutic efficacy of miR-101 via tail vein delivery to an orthotopic liver implanted HCC model of mouse. Lent-miR-101, control lent-miR-ctr and physiological saline (NaCl) was administered, respectively, to mice by tail vein at one week after the preparation of the mouse HCC model, 2 times a week for a month. When mice got moribund, mice were euthanized. The liver, the lung and tumor xenograft were assessed.

**Figure 2:**
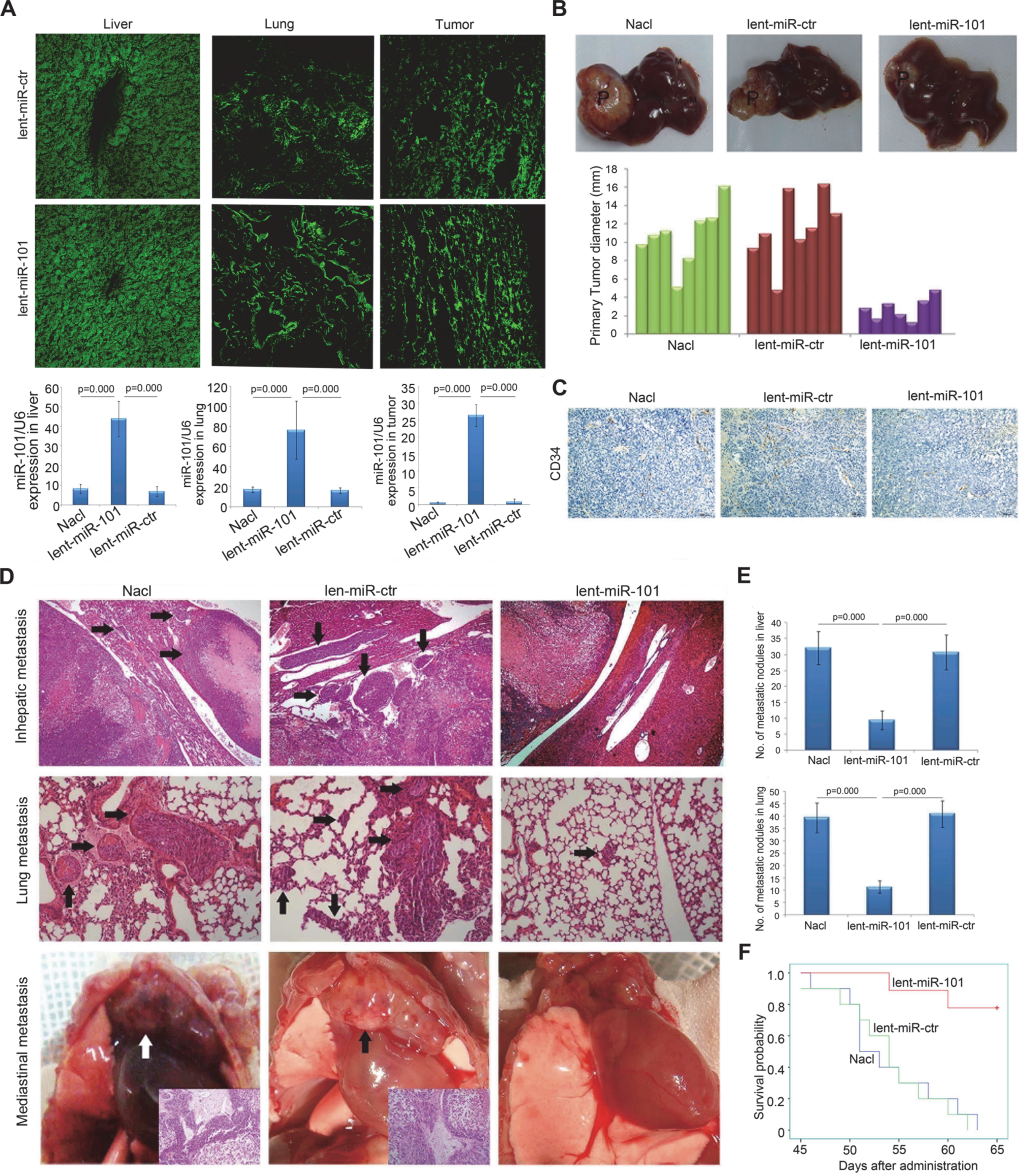
Systemic delivery of lent-miR-101 suppresses tumor growth, angiogenesis and metastasis in the orthotopic liver implanted HCC model of mouse. **A.** Upper panel, laser confocal microscopy showed efficient infection of lentivirus to the liver, the lung and tumor xenografts of mice, as indicated by coGFP expression. Down panel, the levels of miR-101 in the liver, the lung and tumor tissues were significantly higher in lent-miR-101 treated mice than that in both control mice (*P*<0.001). Data is presented as mean± SE. **B.** Representative tumor xenografts in the liver of mice in different (NaCl, lent-miR-ctr and lent-miR-101) treated groups. P, primary tumor; M, metastatic nodule. The average size of primary tumors in the liver was 10.75±3.25 mm in diameter in NaCl treated group (*n* = 8), 11.51±3.71 mm in lent-miR-ctr treated group (*n* = 8), and 2.78±1.25 mm in lent-miR-101 treated group (*n* = 7). **C.** Representative images of microvessel density (MVD) of implanted primary tumor examined by IHC staining of CD34 in lent-miR-101 treated and 2 control groups. **D.** Representative images of metastatic nodules in the liver, in the lung and in the mediastinum of mice in different treatment groups (indicated by arrows). **E.** The mean number of metastasis in lenti-miR-101 treated group (liver: 9.4±2.9; lung: 11.3±2.5, *n* = 7) was significantly larger than that in control NaCl (liver: 32.1±5.1; lung: 39.4±6.0, *n* = 8) and lent-miR-ctr (liver: 30.8±5.4; lung: 40.9±5.4, *n* = 8) treated groups (*P*<0.01). Data is presented as mean± SE. **F.** The difference in survival time of mice between the lenti-miR-101 group and 2 control (NaCl and lent-miR-ctr) groups was statistically significant (*P*<0.05).

Firstly, we observed that the levels of coGFP in the liver, the lung and tumor tissues of lent-miR-101 treated mice were equivalent to that in lent-miR-ctr treated mice, exhibiting over 90% infection efficiency (Fig. 2A, upper panel). But the expression levels of miR-101 in the liver, the lung and tumor tissues were significantly higher in lent-miR-101 treated mice than that in both control mice (p<0.0001, Fig. 2A, down panel). Meanwhile, the administrations of lent-miR-101 and lent-miR-ctr did not cause acute liver toxicity, as demonstrated by the maintenance of normal levels of serum markers of liver function (S1 Table) and an absence of overt histological evidence of toxicity (S2 Fig.).

Next, we found that mice in control groups developed larger sized primary tumors than that in lent-miR-101 treated mice (Fig. 2B, p<0.01). Moreover, we assessed the microvessel density (MVD) of tumors by Immunohistochemistry (IHC) staining of CD34[23] . The MVD-CD34 of tumors in lent-miR-101 group (mean, 18; range, 9–46) was significantly smaller than that in both lent-miR-ctr (mean, 41; range, 25–69) and NaCl (mean, 43; range, 22–78) treated groups (p<0.01, Fig. 2C). Furthermore, we examined that the number of intrahepatic and pulmonary metastatic nodules was dramatically decreased in lent-miR-101 treated group compared to that in both control groups (p<0.01, Fig. 2D and 2E), and the mean survival time in lent-miR-101-treated mice was significantly longer than that in the control mice (p<0.05, Fig. 2F).

### MiR-101 directly targets *ROCK2* 3’UTR

**Figure 3:**
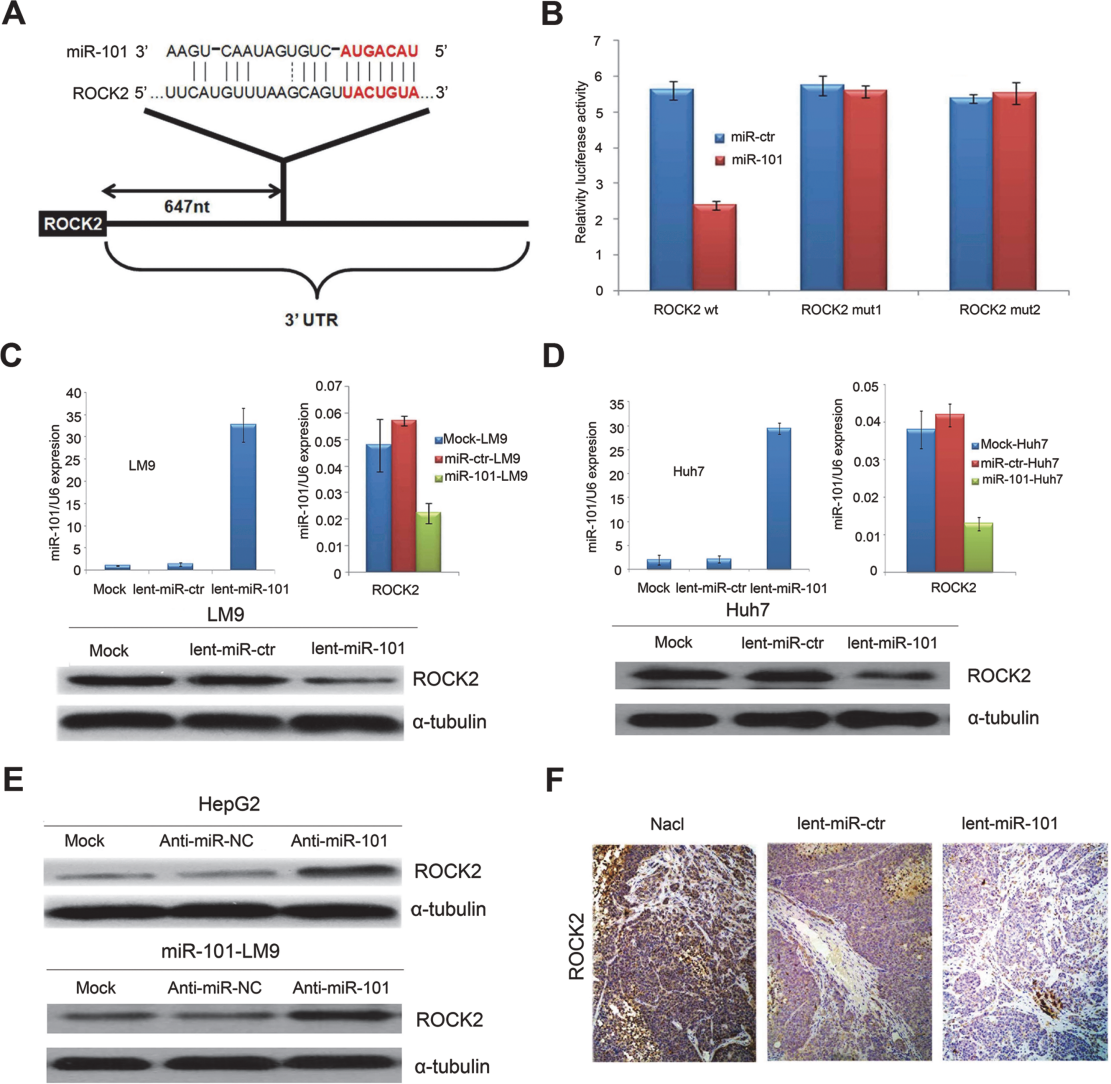
*ROCK2* is the target of miR-101. **A.** Schematic of predicted miR-101-binding sites in the 3′UTR of *ROCK2*. **B.** MiR report constructs containing a wild-type and 2 mutated ROCK2 3’UTR were transfected into LM9 cells, respectively. Relative repression of firefly luciferase expression was standardized to a transfection control. The reporter assays were performed 3 times with essentially identical results. **C.** Left, the levels of miR-101 by Real-time PCR in the lenti-miR-101 and control MOCK and lent-miR-ctr treated LM9 cells. Right, real-time PCR examination of mRNA levels of *ROCK2* between the lenti-miR-101 and control lent-miR-ctr treated LM9 cells. LM9 cells were infected with lent-miR-ctr or lent-miR-101 for 72 hours. Down, ectopic overexpression of miR-101 by lenti-miR-101 reduces the levels of *ROCK2* proteins in LM9 cells, as compared to that in both MOCK and lent-miR-ctr treated LM9 cells. **D.** Left, the levels of miR-101 by Real-time PCR in the lenti-miR-101 and mock and lent-miR-ctr treated Huh7 cells. Right, real-time PCR examination of mRNA level of *ROCK2* between the lenti-miR-101 and control lent-miR-ctr treated Huh7 cells. Huh7 cells were infected with lent-miR-ctr or lent-miR-101 for 72 hours. Down, ectopic overexpression of miR-101 by lenti-miR-101 reduces the levels of *ROCK2* proteins in Huh7 cells, as compared to that in both Mock and lent-miR-ctr treated Huh7 cells. **E.** Upper, protein expression of *ROCK2* is up-regulated in HCC HepG2 cells after the down-regulation of miR-101 by anti-miR-101, as compared to that in control Mock and anti-miR-NC HepG2 cells. Down, protein expressions of *ROCK2* is up-regulated in lent-miR-101-LM9 cells after the down-regulation of miR-101 by anti-miR-101, as compared to that in control anti-miR-NC cells. **F.** IHC staining showing down-regulated expressions of ROCK2 in HCC tissues of mice treated with systemic delivery of lent-miR-101, as compared to that treated with NaCl or lent-miR-ctr.

In our study, the putative targets of miR-101 were predicted using target prediction programs, miRanda and TargetScan. We evaluated that besides the target genes of *EZH2, COX2, STMN1, MCL-1* and *FOS* identified previously [11–13,17,18], the 3’-UTR of *ROCK2* mRNA contains a complementary site for the seed region of miR-101, the *ROCK2* gene was an additional potential target of miR-101 (Fig. 3A).

To verify whether or not *ROCK2* is a direct target of miR-101, *ROCK2* 3’-UTR (Fig. 3A) and two mutants containing the miR-101 binding sites were cloned downstream of the luciferase open reading frame. These reporter constructs were used to cotransfect HCC LM9 cells. The luciferase activity assays showed that increased expression of miR-101 upon infection significantly affected the luciferase expressions of *ROCK2* in LM9 cells. Conversely, when we performed luciferase assays using a plasmid harboring the 3’-UTR of *ROCK2* mRNA, in which the binding sites for miR-101 were inactivated by site-directed mutant genesis, the luciferase activities of mutant reporters were unaffected by the simultaneous infection of miR-101 (Fig. 3B).

In addition, the mRNA and protein levels of *ROCK2* were all substantially reduced after miR-101 overexpression in LM9 and Huh7 cells (Fig. 3C and 3D). On the other hand, knocking down miR-101 in HepG2 and miR-101-LM9 cells, dramatically increased protein levels of *ROCK2* (Fig. 3E). Furthermore, IHC staining showed that ROCK2 expressions were down-regulated in HCC tissues of mice treated with systemic delivery of lent-miR-101 (Fig. 3F). At the same time, we also confirmed that *STMN1* and *COX2* are the other targets of miR-101 in HCC (S3–S4 Fig.).

### Ectopic expression of miR-101 inhibits HCC cell motility, invasion and epithelial-mesenchymal transition (EMT) in vitro

**Figure 4:**
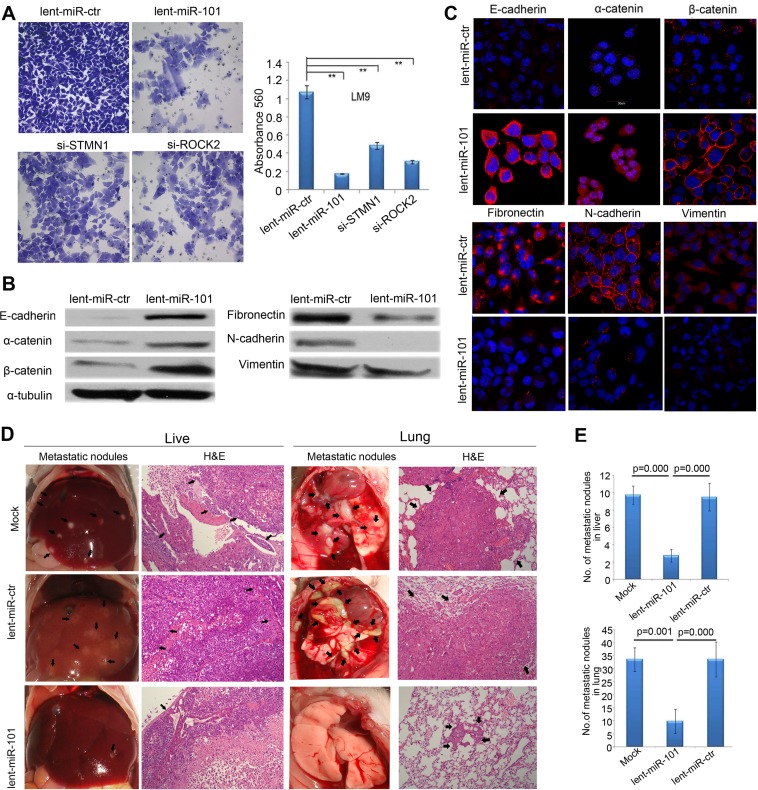
Enforced overexpression of miR-101 inhibits HCC LM9 cells invasion and EMT *in vitro* and reduces metastasis *in vivo*. **A.** The invasive properties of HCC LM9 cells transfected with lent-miR-ctr, lent-miR-101, si-S*TMN1*, and si-*ROCK2* were analyzed by an invasion assay using a Matrigel Invasion Chamber. Migrated cells were plotted as the average number of cells per field of view from 3 independent experiments (**, *P*<0.01). **B.** Expression levels of the epithelial markers E-cadherin, α-catenin and β-catenin and the mesenchymal markers fibronectin, N-cadherin and vimentin were analyzed by Western blot between lent-miR-101 and control lent-miR-ctr LM9 cells. **C.** IF staining (red signal) showing that the Epithelial markers E-cadherin, α-catenin, β-catenin were up-regulated and mesenchymal markers fibronectin, N-cadherin and vimentin were down-regulated in lent-miR-101 treated LM9 cells, as compare to that in lent-miR-ctr cells. **D.** The *in vivo* effects of miR-101 on HCC cell metastasis using an experimental metastasis assay, in which lent-miR-101, control lent-miR-ctr and mock LM9 cells were injected into the tail vein of SCID mice, respectively. Metastatic tumor growth in the liver and in the lung was assessed. Representative metastatic nodules and H&E staining of metastatic tumors in the liver and in the lung are indicated by arrows. **E.** The number of metastatic nodules in the liver and in the lungs of mice (*n* = 8 per group) 8 weeks after tail vein injection of let-miR-101 LM9 cells (mean±SE, liver: 2.8±0.8, lung: 9.8±4.7), mock LM9 cells (mean±SE, liver: 9.8±1.0, lung: 33.5±4.6) and lent-miR-ctr LM9 cells (mean±SE, liver: 9.5± 1.6, lung: 33.5± 6.7).

The Matrigel invasion and Wound healing assays demonstrated that miR-101 overexpression substantially decreased both LM9 and Huh7 HCC cells motility and invasive capability (Fig. 4A; S5–S6A Fig.). Moreover, after miR-101 overexpression in LM9 and Huh7 lines, the expression levels of all tested epithelial markers (E-cadherin, α-catenin and β-catenin) increased, while the levels of mesenchymal markers (fibronectin, N-cadherin and vimentin) decreased (Fig. 4B and 4C; S6B–S6C Fig.).

### Overexpression of miR-101 in HCC cells suppresses metastasis in vivo

We further evaluated the *in vivo* effects of miR-101 overexpression on HCC cell metastasis using an experimental *in vivo* metastasis assay. As demonstrated in Fig. 4D and 4E, the numbers of micrometastatic lesions in the liver and the lungs of mice were markedly reduced in lent-miR-101-LM9 group, as compared to that in the control groups.

### MiR-101 inhibits RhoA/Rac1 GTPase in HCC cells

**Figure 5:**
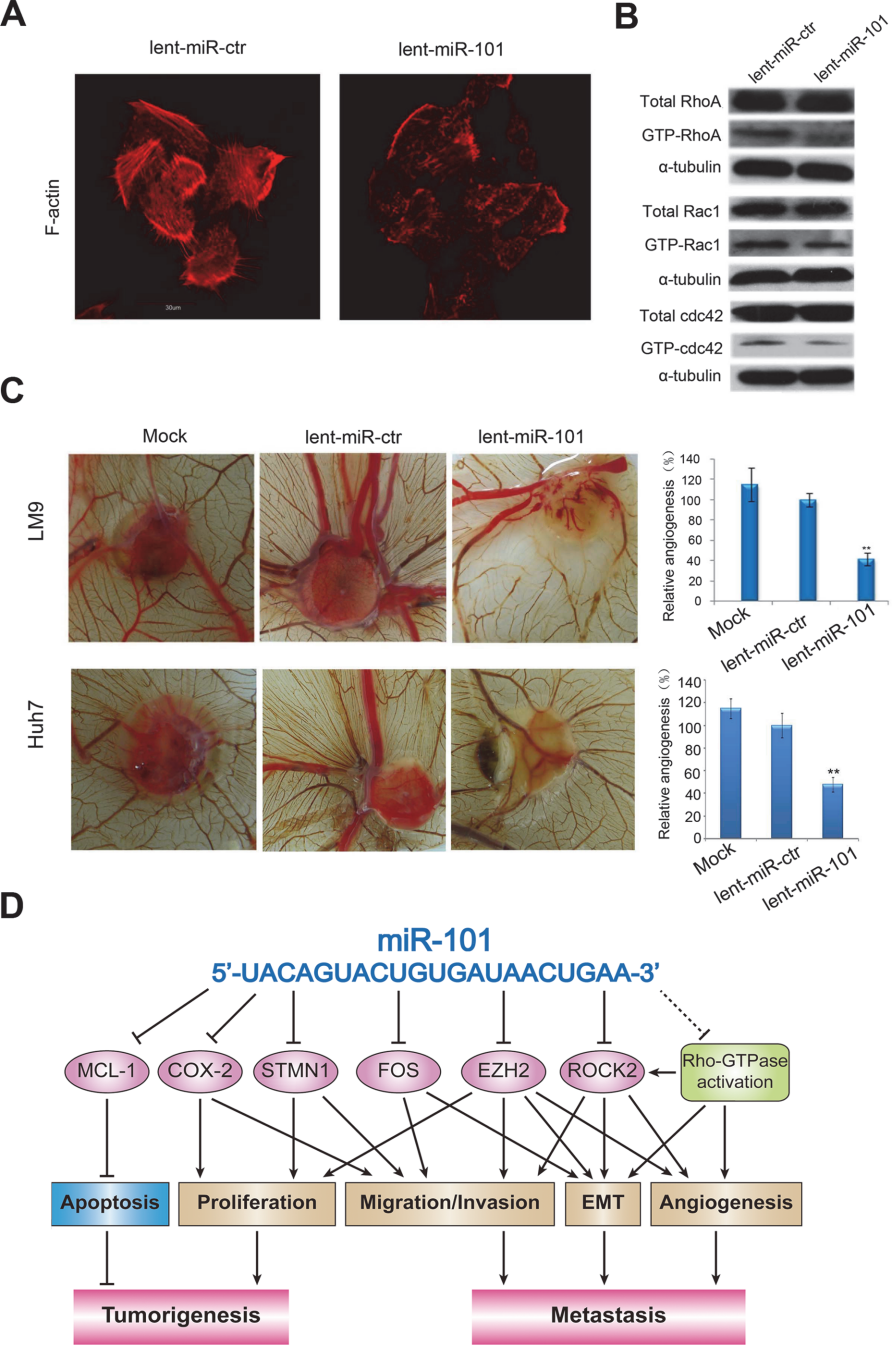
Ectopic overexpression of miR-101 inhibits stress fiber formation *in vitro* and angiogenesis in a CAM model, and a proposed regulatory loop of miR-101 in HCC tumorigenesis and metastasis. **A.** Staining for F-actin demonstrated that the stress fiber formation in lent-miR-101 treated LM9 cells decreased when compared with that in lent-miR-ctr LM9 cells. **B.** Total and active forms of Rho-GTPases, including RhoA, Rac1, and cdc42 were compared between lent-miR-ctr and lent-miR-101 treated LM9 cells by Western blot analysis. GTP-bound (active) forms of RhoA, Rac1, and cdc42 were pulled down and examined by Western blot using corresponding antibodies. Active forms of RhoA, Rac1 and Cdc42 were lower in lent-miR-101 LM9 than that in lent-miR-ctr LM9 cells. **C.** Lent-miR-101 LM9 and Huh7 cells inhibits angiogenesis in a CAM model. Left panel, representative plugs from different (Mock, lent-miR-ctr and lent-miR-101 LM9 and Huh7 cells) treated groups. Right panel, the number of blood vessels was counted from 6 replicate experiments, and normalized to that of the lent-miR-ctr group as relative angiogenesis. The data were mean±SD. ** indicates the significant change with *P*<0.01. **D.** A proposed model in which miR-101 inhibits tumorigenesis and metastasis of HCC by repression of multiple target genes and tumor activators.

It is known, during tumor invasion and metastasis, changes in Rho-GTPase activity often lead to subsequent reorganization of actin cytoskeleton[4,24]. We investigated if miR-101 modifies HCC cell cytoskeleton rearrangement and inhibits Rho-GTPase. The F-actin staining showed that the stress fiber was observed in the control lent-miR-ctr-LM9 and lent-miR-ctr-Huh7 cells, but not in lent-miR-101-LM9 and lent-miR-101-Huh7 cells (Fig. 5A and S7A Fig.) and meanwhile, a lower level of GTP-RhoA, GTP-Rac1 and GTP-cdc42 was examined in lent-miR-101-LM9 and lent-miR-101-Huh7 cells as compared to that in lent-miR-ctr-LM9 and lent-miR-ctr-Huh7 cells (Fig. 5B and S7B Fig.).

### Overexpression of miR-101 in HCC cells inhibits angiogenesis in a chicken chorioallantoic membrane (CAM) model

To test the function of miR-101 in regulating angiogenesis, we also examined the effect of lent-miR-101-LM9 and lent-miR-101-Huh7 cells on angiogenesis in a CAM model. The results showed that ectopic overexpression of miR-101 in LM9 and Huh7 cells dramatically suppressed the angiogenesis *in vivo* (Mock *versus* lent-miR-ctr, and *versus* lent-miR-101 LM9 cells were 112%±12.1% *versus* 98.67%±8.84%, and *versus* 46.33%±9.67%, and mock *versus* lent-miR-ctr, and *versus* lent-miR-101 Huh7 cells were 115%±8.6% versus 100%±10.8%, and versus 48%±6.3%, Fig. 5C).

## Discussion

It is known that most tumors are characterized by globally decreased expression of miRNAs[6] and enforced down-regulation of certain specific miRNAs may enhance cellular transformation and tumorigenesis[25]. Recently, accumulative evidences suggested that therapeutic delivery of certain miRNA(s) has a unique advantage in clinical use, since an individual miRNA may influence the cellular behavior through the regulation of multiple target genes and networks[26]. We and other groups previously identified a frequently down-regulated miRNA, miR-101, in various solid tumors including HCC [11,12]. In the present study, our initial Quantitative PCR demonstrated that in a large cohort of HCC patients, all HCCs with distant metastasis had a low level of plasma miR-101, and it was associated closely with an advanced clinical stage and predicted poor prognosis. Next, in an orthotopic liver implanted HCC model of mouse, we clearly showed that systemic delivery of lenti-miR-101 not only induced a dramatic suppression of tumor growth in the liver, but also substantially diminished HCC intrahepatic metastasis and distant metastasis to the lung and to the mediastinum, resulting in a dramatic suppression of HCC in mice without a measurable toxicity. The results reveal that therapeutic delivery of lenti-miR-101 in mouse potently inhibits HCC development/metastasis *in vivo* and thereby, establishing a principle that miR-101 may be useful as an effective anti-HCC agent through its ability to broadly suppress HCC cells tumorigenicity and invasiveness.

In this study, we chose a lentivirus-based vector system as miR-101 delivery vehicle, since it is an attractive platform for regulatory gene delivery [27,28]. Importantly, the safety of using the lentiviral vector in preclinical research and clinical trials is a minimal concern and providing a therapeutic benefit for the patients [29–31]. In our mouse HCC model of the present study, we did observe that systemic delivery of lent-miR-101 exhibited an over 90% infective efficiency and high expression levels of miR-101 in the liver, the lung and tumor tissues of mice without toxicity, indicating that lentivirus provides an effective and nontoxic means to deliver miRNAs to mouse. Overall, our data hereby provide a basis for the concept that the systemic administration of miR-101 mediated by lentivirus might be a clinically viable anti-HCC therapeutic strategy. These results prompt us to further explore the functions and excise molecular mechanisms of miR-101 in the development and/or progression of HCC.

Firstly, we observed that systemic delivery of lent-miR-101 in the mouse HCC model substantially inhibited tumor angiogenesis. In a CAM Model, we also demonstrated that enforced expression of miR-101 in HCC cells could diminish angiogenesis. In addition, ectopic overexpression of miR-101 in HCC cells not only suppressed cell motility, invasion and EMT, but also blocked the formation of cells stress fiber. In an experimental *in vivo* metastasis model of SCID mouse, we further showed that the tail-vein-injection of miR-101-overexpressing HCC cells led to a significant decrease in the number of metastatic lesions in the liver and in the lung. These data, collectively, strongly supported that miR-101 plays a crucial tumor suppressive role in the control of HCC aggressive process.

We know that miRNAs exert their functions through regulating downstream target gene(s). The oncogenes, *EZH2* and *COX2*, were two targets of miR-101 first identified in 2008 [17,18] and they were confirmed in our HCC cells of the present study (S4 Fig. and S8 Fig.). *EZH2* contributes to cancer metastasis via regulation of actin-dependent cell adhesion and migration [32] and implicated in EMT [33] and angiogenesis induction [34]. In human HCCs, we previously reported that both *EZH2* and *COX2* are frequently overexpressed in HCCs and positively correlated with high aggressive and/or poor prognostic phenotypes [21,35]. In addition, *COX-2* inhibitors could prevent HCC cell growth both in vitro and in vivo [35,36]. These data suggest that *EZH2* and *COX2* are two important targets of miR-101 to suppress HCC. In 2009, another direct target gene of miR-101, *MCL-1*, was identified by our group and enforced expression of miR-101 in HCC cells could dramatically decrease *MCL-1* levels and thus, promoting apoptosis to suppress tumorigenicity [11]. Almost at the same time, Li et al [12] showed that miR-101 significantly repressed the abilities of HCC cell migration and invasion by targeting the *FOS* oncogene. And FOS can induce EMT in mammary epithelial cells [37]. More recently, Xu and colleagues [13] reported that miR-101 could inhibit autophagy and enhance cisplatin-induced apoptosis in HCC cells by targeting *STMN1. STMN1* is a key microtubule-regulatory protein and associated positively with HCCs vascular invasion, intrahepatic metastasis and advanced clinical stage [38,39]. In a series of *in vitro* and *in vivo* experiments of our present study, we not only confirmed that *STMN1* is a target of miR-101 (S2 Fig.), but also identified a novel direct target of miR-101, *ROCK2*, in HCC. Overexpression of *ROCK2* was frequently examined in HCCs and it could induce EMT [4] and a more aggressive biological behavior [40]. These data suggest that miR-101 could enhance its inhibiting effects on HCC by targeting an additional oncogene *ROCK2*.

Cytoskeletal reorganization exemplified by the formation of stress fiber bundling arrays is essential for the contractile motion of cancer cells [24]. In addition, the RhoA/ROCK signaling plays important roles in multiple aspects of VEGF-mediated angiogenesis [41]. Among the members of the Rho family, *RhoA, Rac1* and *Cdc42* are 3 representative members defined in modulating the actin cytoskeleton [42] and may lead to EMT [43], and RhoA activation may induce the formation of stress fibers [44]. In our study, we found that after miR-101 overexpression, the formation of stress fiber in HCC cells was inhibited, and concurrently, the levels of active *RhoA, Rac1* and *Cdc42* were all reduced. As a result of our collective present data, we therefore propose herein a molecular model, in which miR-101 broadly abrogates HCC tumorigenesis and metastasis by a direct suppression of multiple molecular targets and an inactivation of the RhoA/ROCK pathway (Fig. 5D).

To sum up, herein, we report, for the first time, an essential role for systemic delivery of lent-miR-101 in the efficient therapy of HCC in a mouse model, and the use of lentivirus vector has a unique advantage to enhance a transduction and therapeutic abundance of miRNA *in vivo* without toxic effects. Furthermore, functional and/or mechanistic studies of miR-101 as provided in this report, suggest a critical role of miR-101 in the control of HCC cells cytoskeletal reorganization, EMT, invasiveness and angiogenesis, resulting in a potent abrogation of HCC development and progression by means of a “one-hit/multiple-targets” mechanism.

## Materials and Methods

### Tissue specimens and cell cultures

One hundred and sixty-three cases of blood samples from patients with HCC were obtained from the residue of patient blood samples for the purpose of clinical diagnosis in the Clinical Laboratory of Sun Yat-Sen University Cancer Center, Guangzhou, China, between July 2005 and June 2010. The HCC cases selected were based on distinctive pathologic diagnosis, availability of blood specimens, follow-up data, and had not received previous local or systemic treatment. In this HCC cohort, 147 cases were HCCs without distant metastasis, 16 HCCs had distant metastasis. Relevant corresponding clinical data of HCC patients were detailed in Table 1. In addition, 50 cases of plasma samples from human healthy donors were utilized as control. Blood samples were processed and plasma was frozen within 2 hours of the blood draw. In the present study, the informed consents of participants have not been conducted and given, since 1) the privacy and personal identity information of all participants were protected, i.e., all the data were analyzed anonymously, 2) all the blood samples were not and will not be used for any other purpose and 3) the waiver of informed consent did not and will not have adverse effect on the rights and health of the participants. This study was approved by the Institute Research Medical Ethics Committee at Sun Yat-Sen University Cancer Center.

HCC cell lines Hep3B, Bel-7402, SMMC-7721 and MHCC-LM9 were cultured in RPMI1640 medium with 10% newborn calf serum. Immortalized normal liver cell line MIHA, human embryonic kidney cell 293FT and HCC Huh-7 and HepG2 lines in DMEM were cultured with 10% fetal bovine serum.

### RNA isolation and quantitative real-time PCR

RNA was isolated from 400μl plasma using the mirVana PARIS kit (Ambion, Carlsbad, CA) following the manufacturer’s protocol. To allow for normalization of sample to sample variation in the RNA isolation step, synthetic cel-miR-39 was added to each sample as described by Mitchell et al [45]. These samples were processed under the exactly same conditions.

To ensure the quality of RNA from plasma, we examined the levels of miR-16, a miRNA displays the higher stability in plasma [46], in 163 HCC patients and 50 healthy donors. The results showed that no significant difference was examined in terms of the plasma levels of miR-16 between healthy donors and HCC patients (S1C Fig.).

Total RNA from cell lines and tissues was extracted with TRIzol reagent (Invitrogen, Carsbad, CA). cDNA was synthesized with the PrimeScript RT reagent Kit (Promega, Madison, WI). Real-time PCR was carried out using an ABI 7900HT Fast Real-time PCR system (Applied Biosystems, Foster City, CA) according to the manufacturer’s recommended conditions. The primer sequences are provided in S2 Table.

MicroRNA measurement by real-time PCR was performed in duplicate using Taqman universal PCR kit and miR-101 and RUN6B/ cel-miR-39 probe (Applied Biosystems, Foster City, CA), in scaled-down (5μL) reaction volumes using 2.5μL TaqMan 2× Universal PCR Master Mix with No AmpErase UNG, 0.25μL miRNA-specific primer/probe mix, and 2.25μL diluted RT product per reaction. At the end of the PCR cycles, melting curve analyses as well as electrophoresis of the products on 3.0% agarose gels were performed in order to validate the specific generation of the expected PCR product. Each sample was run in duplicates for analysis. △Ct was calculated by subtracting the Ct values of U6/ cel-miR-39 from the Ct values of the miRNA of interest. △△Ct was then calculated by subtracting △Ct of the control from △Ct of disease. Fold change of gene was calculated by the equation 2^−△△Ct^ .

### Lentivirus production and HCC cell infection

Virus particles were harvested 48h after pCDH-CMV-miR-101-coGFP or pCDH-CMV-coGFP (System Biosciences, CA) transfection with the packaging plasmid pRSV/REV, pCMV/VSVG and pMDLG/pRRE into 293FT cells by using Lipofectamine 2000 reagent (Invitrogen). Lentivirus-miR-101-coGFP (lent-miR-101) and lentivirus-miR-ctr-coGFP (lent-miR-ctr) were condensed and purified for 10^8^ MOI/200μl. Next, LM9 and Huh-7 HCC cells were infected by lent-miR-101 and lent-miR-ctr, respectively, to construct the stable miR-101-expressing and control HCC cells.

### Oligonucleotide transfection

MiR-101 inhibitor was synthesized by Genepharma (Shanghai, China). The sequence of miR-101 inhibitor is UUCAGUUAUCACAGUACUGUA. SiRNA duplex oligonucleotides targeting human *ROCK2* mRNA 5’-CAGAAGCGTTGTCTTATGCAA-3’, targeting human *STMN1* mRNA 5’-AAGAGAAACUGACCC-ACAAdTdT-3’ and targeting COX2 mRNA 5’-GCUGGGAAGCCUUCUCUAAdTdT-3’ were synthesized by Ribobo (Guangzhou, China). Oligonucleotide transfection was performed with Lipofectamine 2000 reagents (Invitrogen).

### Luciferase reporter assay

The putative miR-101 binding sites at the 3’-UTRs of *ROCK2, STMN1* and *COX2* mRNAs were cloned downstream of the cytomegalovirus (CMV) promoter in a pMIR-REPORT vector (Ambion). Two mutant constructs were generated by either deletion or mutations. The primers used are shown in S2 Table.

The firefly luciferase construct was cotransfected with a control Renilla luciferase vector into LM9 cells in the presence of either lent-miR-101 or lent-miR-ctr. Dual luciferase assay (Promega) was performed 48 hours after transfection. The experiments were performed independently in triplicate.

### Wound healing and invasion assays

Cell migration was assessed by measuring the movement of cells into a scraped, cellular area created by a 200-μl pipette tube, and the spread of wound closure was observed after 48 hours and photographed under a microscope. We measured the fraction of cell coverage across the line for migration rate. For invasion assays, 10^5^ cells were added to a Matrigel^TM^ Invasion Chamber (BD Biosciences, Becton Dickson Labware, Flanklin Lakes, NJ) present in the insert of a 24 well culture plate. Fetal bovine serum was added to the lower chamber as a chemoattractant. After 48 hours, the non-invading cells were gently removed with a cotton swab. Invasive cells located on the lower side of the chamber were stained with crystal violet, air dried and photographed. For colorimetric assays, the inserts were treated with 150μl 10% acetic acid and the absorbance was measured at 560 nm using a spectrophotometer (Spectramax M5).

### Western blot analysis and Rho-GTPase activation assay

Proteins were separated on SDS-PAGE and transferred to nitrocellulose membrane (Bio-Rad). The membrane was blocked with 5% non-fat milk and incubated with the corresponding mouse anti-ROCK2, EZH2, COX2, E-cadherin, α-catenin, β-catenin, N-cadherin, fibronectin, vimentin (BD Biosciences, 1:1000 dilution), STMN1 and α-tubulin (Santa Cruz Biotechnology, Santa Cruz, CA, 1:1000 dilution) and GAPDH (Cell signaling Technology, Beverly, MA, 1:500 dilution) monoclonal antibodies. The proteins were detected with enhanced chemiluminescence reagents.

PAK1 PBD-agarose (for isolating Rac1-GTP and cdc42-GTP) and rhotekinagarose (for isolating Rho-GTP) (Upstate Biotechnology, Lake Placid, NY) were used to pull down the GTP-bound form of Rho-GTPase according to the manufacturer’s manual. The levels of active Rac1, cdc42 and RhoA were detected by Western blot using specific polyclonal anti-Rac1 (1:1000), anti-cdc42 (1:1000) and anti-RhoA (1:1000) antibodies (Cell Signaling Technology, Beverly, MA).

### Immunofluorescence (IF)

For the IF studies, cells were fixed with 4% paraformaldehyde in phosphate-buffered saline and permeabilized with 0.2% Triton X-100 in phosphate-buffered saline. Fixed cells were incubated with 1:2000 fluorescein isothiocyanate-conjugated phalloidin (Sigma, St. Louis, MO) or antibodies as indicated. Cells were counterstained with 4, 6-diamidino-2-phenylindole (DAPI) (Calbiochem, San Diego, CA) and imaged with a confocal laser-scanning microscope (Olympus FV1000, Tokyo, Japan).

### Systemic delivery of lent-miR-101 to an orthotopic liver implanted HCC model of mouse

Male nude mice (BALB/C-nu/nu), 4–5 weeks old, 15–20 g, were obtained from the Center of Animal Control of Guangdong Province and maintained in an Animal Biosafety Level 3 Laboratory at the Animal Experiment Center of Sun Yat-Sen Cancer Center. We did the experiment 3–5 days after delivery of the mice to allow them to adapt to the environment.

First, to establish an orthotopic liver implanted HCC model of mouse, 2 × 10^6^ populations of LM9 cells, were injected subcutaneously into the flanks of BALB/C-nu/nu athymic nude mice. After 2 weeks, the subcutaneous tumors were resected and diced into 1 mm^3^ cubes, which were then implanted into the liver of mice. After the model construction, the mice have been intraperitoneally injected gentamycin in order to prevent abdominal infection, one time one day for three days. Then lentivirus-miR-101-coGFP (lent-miR-101), lentivirus-miR-ctr-coGFP (lent-miR-ctr) and physiological saline (NaCl) were administered at a dose of 10^8^ MOI per animal by tail vein injection (200 μl total volume) using a 30 gauge ultra-fine insulin syringe at a week after the model construction, 2 times a week for a month. Meanwhile, the general health status of the nude mice was observed everyday, including food intake, activity, and any abnormalities such as diarrhea and dehydration. The body weight was measured every 3 days. When animals became moribund, mice were euthanized by the cervical dislocation method. The liver and the lungs were removed and fixed with phosphate-buffered formalin. Subsequently, consecutive tissue sections were made for each block of the liver and the lung. The numbers of the intrahepatic and pulmonary metastatic nodules in the liver and the lung were carefully examined. All experimental procedures involving animals were are accordant with the Guidelines for the Care and Use of Laboratory Animals (NIH publications Nos. 80–23, revised 1996) and the laboratory animal ethics committee of Sun Yat-Sen University Cancer Center.

### Immunohistochemistry (IHC)

The tissue blocks were cut into 5-μm sections and processed for IHC in accordance with a previously described protocol. [21]

### Experimental in vivo metastasis model

Eight 4-week-old male SCID-Beige mice in each experimental group were injected with lent-miR-101-LM9, lent-miR-ctr-LM9 and mock-LM9 cells separately. Briefly, 1×10^5^ cells were injected intravenously through tail vein into each SCID mouse in a laminar flow cabinet. Six weeks after cell injection, mice were sacrificed and examined.

### Angiogenesis assay in a chicken chorioallantoic membrane (CAM) model

Fertilized chicken eggs were purchased from institute of zoo techniques and veterinary science (Guangzhou, China), and incubated at 37℃ with 70% humidity for 8 days. Lent-miR-101, lent-miR-ctr and mock LM9 cells were re-suspended in PBS buffer solution. Cells (2×10^6^ cells, 15 μl) were mixed with equal volume of Matrigel (BD Biosciences). Aliquots (2×10^6^ cells, 30 μl) of the mixture were then applied onto the CAM of 9-day-old embryos. The area around the implanted Matrigel was photographed 4 days after the implantation, and the number of blood vessels was obtained by counting the branching of blood vessels. Assays for each treatment were carried out using 6 chicken embryos.

### Statistical analysis

Statistical analysis was performed using a SPSS software package (SPSS Standard version 16.0, SPSS Inc.). The ROC curve analysis was applied to define a cutoff score for plasma miR-101 level by a 0, 1- criterion[47]. Briefly, the sensitivity and specificity for the outcome (survival status) were plotted to create a ROC curve. The score localized closest to the point (i.e., 0.0, 1.0) at the maximum sensitivity and specificity was selected as the cutoff score to determine the greatest number of tumors that were correctly classified as having or not having the outcome. Data derived from cell line experiments are presented as mean ±SE and assessed by a Two-tailed Student’s *t* test. *P* values of <0.05 were considered significant.

### Accession numbers

Human miR-101, MI0000937; human ROCK2, Homo sapiens ROCK2 NM_004850; Human STMN1, NM_005563.3 Homo sapiens stathmin 1 (STMN1), transcript variant 3, mRNA.

### Ethics approval

This study was approved by the Institute Research Medical Ethics Committee of Sun Yat-Sen University Cancer Center, Guangzhou, China.

## Supporting Information

S1 Fig Analysis of miR-101 levels in human plasma samples by real-time PCR. **A.** The level of mature miR-101 in HBV-negative (*n* = 29) and HBV-positive (*n* = 134) HCC patient’s plasma. **B.** The level of mature miR-101 in HBV-negative (*n* = 5) and HBV-positive (*n* = 10) HCC patient’s plasma with distant metastasis. **C.** CT value of miR-16 in human plasma samples from healthy donors (normal, *n* = 50) and HCC patients (*n* = 163).
(TIF)

S2 Fig No obviously organ-related toxicity was observed. Lent-miR-101 and control lent-miR-ctr was administered, respectively, to mice by tail vein at one week after the preparation of the mouse HCC model, 2 times a week for a month. After the experiment, animals were sacrificed and the organs were fixed in formalin overnight and processed for paraffin embedding. The paraffin-embedded blocks were sectioned and stained by hematoxylin and eosin.
(TIF)

S3 Fig *STMN1* is the target of miR-101. **A.** Schematic of predicted miR-101-binding sites in the 3′UTR of *STMN1*. **B.** MiR report constructs containing a wild-type and 2 mutated *STMN1* 3’UTRs were transfected into LM9 cells, respectively. Relative repression of firefly luciferase expression was standardized to a transfection control. The reporter assays were performed 3 times with essentially identical results. **C.** Upper, real-time PCR examination of mRNA levels of *ROCK2* between the lenti-miR-101 and control lent-miR-ctr treated LM9 cells. LM9 cells were infected with lent-miR-ctr or lent-miR-101 for 72 hours. Down, ectopic overexpression of miR-101 by lenti-miR-101 reduces the levels of *STMN1* protein in LM9 cells, as compared to that in both Mock and lent-miR-ctr treated LM9 cells. **D.** Protein expression of *STMN1* is up-regulated in HCC HepG2 cells after the down-regulation of miR-101 by anti-miR-101, as compared to that in control Mock and anti-miR-NC HepG2 cells. **E.** IHC staining showing down-regulated expressions of *STMN1* in HCC tissues of mice treated with systemic delivery of lent-miR-101, as compared to that treated with NaCl or lent-miR-ctr.
(TIF)

S4 Fig Enforced expression of miR-101 in HCC cell line inhibits the mRNA and protein levels of *COX2*. **A.** Enforced overexpression of miR-101 in LM9 cells decreases endogenous levels of *COX2* protein. LM9 cells were infected with Mock, lent-miR-ctr or lenti-miR-101 for 72 hours. *COX2* expression was assessed by Western blot. **B.** MiR report constructs containing a wild-type and 2 mutated *COX2* 3’UTRs were transfected into LM9 cells, respectively. Relative repression of firefly luciferase expression was standardized to a transfection control. The reporter assays were performed 3 times with essentially identical results. **C.** The mRNA levels of *COX2* in Mock, lent-miR-ctr or lenti-miR-101 LM9 cells examined by Real-time PCR. Lenti-miR-101 decreased the levels of *COX2* mRNA in LM9 cells. **D.** Western blot assay showing protein levels of *COX2* after the treatment of Mock, Anti-miRNC and anti-miR-101 in HepG2 cell line. Anti-miR-101 could increase *COX2* expression in HepG2 cells.
(TIF)

S5 Fig Wound-healing assay demonstrating different cll motilities in lent-miR-ctr, lent-miR-101, si-*STMN1* and si-*ROCK2* treated HCC lines LM9 and Huh7. Ectopic overexpression of miR-101 by infection of lent-miR-101 decreased cell motility in both LM9 and Huh7 cells, compared with that in lent-miR-ctr control cells. Silence of either *ROCK2* or *STMN1* by specific siRNA could partially mimic the inhibiting effect of lent-miR-101 on both HCC cells motilities.
(TIF)

S6 Fig Ectopic overexpression of miR-101 inhibits HCC Huh7 cell invasion and EMT *in vitro*. **A.** The invasive properties of HCC Huh cells transfected with lent-miR-ctr, lent-miR-101, si-*STMN1*, and si-*ROCK2* were analyzed by an invasion assay using a Matrigel Invasion Chamber. Migrated cells were plotted as the average number of cells per field of view from 3 indipendent experiments (**, *P*<0.01). **B.** Expression levels of the epithelial markers E-cadherin, α-catenin, β-catenin and the mesenchymal markers fibronectin, N-cadherin and vimentin were analyzed by Western blot between lent-miR-101 and control lent-miR-ctr treated Huh cells. **C.** IF staining was used to compare expression levels/pattern of epithelial markers and mesenchymal markers (red signal) between the control lent-miR-ctr and lent-miR-101 treated Huh cells. The Epithelial markers E-cadherin, α-catenin, β-catenin were upregulated and mesenchymal markers fibronectin, N-cadherin and vimentin were downregulated in lent-miR-101 treated Huh cells, as compare to that in lent-miR-ctr Huh cells.
(TIF)

S7 Fig Ectopic overexpression of miR-101 inhibits stress fiber formation *in vitro*. **A.** Staining for F-actin demonstrated that the stress fiber formation in lent-miR-101 treated Huh7 cells decreased when compared with that in lent-miR-ctr Huh7 cells. **B.** Total and active forms of Rho-GTPases, including RhoA, Rac1, and cdc42 were compared between lent-miR-ctr and lent-miR-101 treated Huh7 cells by Western blot analysis. GTP-bound (active) forms of RhoA, Rac1, and cdc42 were pulled down and examined by Western blot using corresponding antibodies. Active forms of RhoA, Rac1 and Cdc42 were lower in lent-miR-101 Huh7 than that in lent-miR-ctr Huh7 cells.
(TIF)

S8 Fig Enforced expression of miR-101 in HCC cell line inhibits the mRNA and protein levels of *EZH2*. **A.** Enforced overexpression of miR-101 in LM9 cells decreases endogenous levels of *EZH2* protein. LM9 cells were infected with Mock, lent-miR-ctr or lenti-miR-101 for 72 hours. *EZH2* expression was assessed by Western blot. **B.** The mRNA levels of *EZH2* in Mock, lent-miR-ctr or lenti-miR-101 LM9 cells examined by Real-time PCR. Lenti-miR-101 decreased the levels of *EZH2* mRNA in LM9 cells. **C.** Western blot assay showing protein levels of *EZH2* after the treatment of Mock, Anti-miRNC and anti-miR-101 in HepG2 cell line. Anti-miR-101 could increase *EZH2* expression in HepG2 cells.
(TIF)

S1 Table Main serological parameters of 3 groups of nude mice at the study endpoints.
(DOC)

S2 Table Primers for ROCK2, STMN1 and COX2 3’-UTRs in the luciferase report assay and real-time PCR primers for ROCK2, STMN1, EZH2 and COX2.
(DOC)

## References

[1] Roberts LR (2008) Sorafenib in liver cancer—just the beginning. N Engl J Med 359: 420–422. 10.1056/NEJMe0802241 18650519

[2] Pisani P, Parkin DM, Bray F, Ferlay J (1999) Erratum: Estimates of the worldwide mortality from 25 cancers in 1990. Int. J. Cancer, 83, 18–29 (1999). Int J Cancer 83: 870–873. 10602059 10.1002/(sici)1097-0215(19991210)83:6<870::aid-ijc35>3.0.co;2-9

[3] Calin GA, Croce CM (2006) MicroRNA signatures in human cancers. Nat Rev Cancer 6: 857–866. 10.1038/nrc1997 17060945

[4] Zheng F, Liao YJ, Cai MY, Liu YH, Liu TH, et al. (2012) The putative tumour suppressor microRNA-124 modulates hepatocellular carcinoma cell aggressiveness by repressing ROCK2 and EZH2. Gut 61: 278–289. 10.1136/gut.2011.239145 21672940

[5] Yang X, Zhang XF, Lu X, Jia HL, Liang L, et al. (2013) MicroRNA-26a suppresses angiogenesis in human hepatocellular carcinoma by targeting hepatocyte growth factor-cMet pathway. Hepatology 59(5):1874–85. 10.1002/hep.26941 24259426

[6] Lu J, Getz G, Miska EA, Alvarez-Saavedra E, Lamb J, et al. (2005) MicroRNA expression profiles classify human cancers. Nature 435: 834–838. 10.1038/nature03702 15944708

[7] de Fougerolles A, Vornlocher HP, Maraganore J, Lieberman J (2007) Interfering with disease: a progress report on siRNA-based therapeutics. Nat Rev Drug Discov 6: 443–453. 10.1038/nrd2310 17541417 PMC7098199

[8] Jimenez-Mateos EM, Engel T, Merino-Serrais P, McKiernan RC, Tanaka K, et al. (2012) Silencing microRNA-134 produces neuroprotective and prolonged seizure-suppressive effects. Nat Med 18: 1087–1094. 10.1038/nm.2834 22683779 PMC3438344

[9] Kumar MS, Erkeland SJ, Pester RE, Chen CY, Ebert MS, et al. (2008) Suppression of non-small cell lung tumor development by the let-7 microRNA family. Proc Natl Acad Sci U S A 105: 3903–3908. 10.1073/pnas.0712321105 18308936 PMC2268826

[10] Kota J, Chivukula RR, O’Donnell KA, Wentzel EA, Montgomery CL, et al. (2009) Therapeutic microRNA delivery suppresses tumorigenesis in a murine liver cancer model. Cell 137: 1005–1017. 10.1016/j.cell.2009.04.021 19524505 PMC2722880

[11] Su H, Yang JR, Xu T, Huang J, Xu L, et al. (2009) MicroRNA-101, down-regulated in hepatocellular carcinoma, promotes apoptosis and suppresses tumorigenicity. Cancer Res 69: 1135–1142. 10.1158/0008-5472.CAN-08-2886 19155302

[12] Li S, Fu H, Wang Y, Tie Y, Xing R, et al. (2009) MicroRNA-101 regulates expression of the v-fos FBJ murine osteosarcoma viral oncogene homolog (FOS) oncogene in human hepatocellular carcinoma. Hepatology 49: 1194–1202. 10.1002/hep.22757 19133651

[13] Xu Y, An Y, Wang Y, Zhang C, Zhang H, et al. (2013) miR-101 inhibits autophagy and enhances cisplatin-induced apoptosis in hepatocellular carcinoma cells. Oncol Rep 29: 2019–2024. 10.3892/or.2013.2338 23483142

[14] Iorio MV, Ferracin M, Liu CG, Veronese A, Spizzo R, et al. (2005) MicroRNA gene expression deregulation in human breast cancer. Cancer Res 65: 7065–7070. 10.1158/0008-5472.CAN-05-1783 16103053

[15] Mattie MD, Benz CC, Bowers J, Sensinger K, Wong L, et al. (2006) Optimized high-throughput microRNA expression profiling provides novel biomarker assessment of clinical prostate and breast cancer biopsies. Mol Cancer 5: 24 10.1186/1476-4598-5-24 16784538 PMC1563474

[16] Bottoni A, Zatelli MC, Ferracin M, Tagliati F, Piccin D, et al. (2007) Identification of differentially expressed microRNAs by microarray: a possible role for microRNA genes in pituitary adenomas. J Cell Physiol 210: 370–377. 10.1002/jcp.20832 17111382

[17] Strillacci A, Griffoni C, Sansone P, Paterini P, Piazzi G, et al. (2009) MiR-101 downregulation is involved in cyclooxygenase-2 overexpression in human colon cancer cells. Exp Cell Res 315: 1439–1447. 10.1016/j.yexcr.2008.12.010 19133256

[18] Varambally S, Cao Q, Mani RS, Shankar S, Wang X, et al. (2008) Genomic loss of microRNA-101 leads to overexpression of histone methyltransferase EZH2 in cancer. Science 322: 1695–1699. 10.1126/science.1165395 19008416 PMC2684823

[19] Wang L, Zhang X, Jia LT, Hu SJ, Zhao J, et al. (2013) c-Myc-mediated epigenetic silencing of MicroRNA-101 contributes to dysregulation of multiple pathways in hepatocellular carcinoma. Hepatology. 10.1002/hep.26720 24002871

[20] Hayashi Y, Tsujii M, Wang J, Kondo J, Akasaka T, et al. (2013) CagA mediates epigenetic regulation to attenuate let-7 expression in Helicobacter pylori-related carcinogenesis. Gut 62: 1536–1546. 10.1136/gutjnl-2011-301625 22936674

[21] Cai MY, Tong ZT, Zheng F, Liao YJ, Wang Y, et al. (2011) EZH2 protein: a promising immunomarker for the detection of hepatocellular carcinomas in liver needle biopsies. Gut 60: 967–976. 10.1136/gut.2010.231993 21330577

[22] Wei X, Xiang T, Ren G, Tan C, Liu R, et al. (2013) miR-101 is down-regulated by the hepatitis B virus x protein and induces aberrant DNA methylation by targeting DNA methyltransferase 3A. Cell Signal 25: 439–446. 10.1016/j.cellsig.2012.10.013 23124077

[23] Weidner N (1995) Intratumor microvessel density as a prognostic factor in cancer. Am J Pathol 147: 9–19. 7541613 PMC1869874

[24] Wang Y, Senoo H, Sesaki H, Iijima M (2013) Rho GTPases orient directional sensing in chemotaxis. Proc Natl Acad Sci U S A 110: E4723–4732. 10.1073/pnas.1312540110 24248334 PMC3856778

[25] Mori M, Triboulet R, Mohseni M, Schlegelmilch K, Shrestha K, et al. (2014) Hippo Signaling Regulates Microprocessor and Links Cell-Density-Dependent miRNA Biogenesis to Cancer. Cell 156: 893–906. 10.1016/j.cell.2013.12.043 24581491 PMC3982296

[26] Chiou GY, Chien CS, Wang ML, Chen MT, Yang YP, et al. (2013) Epigenetic regulation of the miR142-3p/interleukin-6 circuit in glioblastoma. Mol Cell 52: 693–706. 10.1016/j.molcel.2013.11.009 24332177

[27] Bailey SN, Ali SM, Carpenter AE, Higgins CO, Sabatini DM (2006) Microarrays of lentiviruses for gene function screens in immortalized and primary cells. Nat Methods 3: 117–122. 10.1038/nmeth848 16432521

[28] Schambach A, Zychlinski D, Ehrnstroem B, Baum C (2013) Biosafety features of lentiviral vectors. Hum Gene Ther 24: 132–142. 10.1089/hum.2012.229 23311447 PMC3581032

[29] Cavazzana-Calvo M, Payen E, Negre O, Wang G, Hehir K, et al. (2010) Transfusion independence and HMGA2 activation after gene therapy of human beta-thalassaemia. Nature 467: 318–322. 10.1038/nature09328 20844535 PMC3355472

[30] Flight MH (2013) Trial watch: Clinical trial boost for lentiviral gene therapy. Nat Rev Drug Discov 12: 654 10.1038/nrd4111 23989781

[31] Aiuti A, Biasco L, Scaramuzza S, Ferrua F, Cicalese MP, et al. (2013) Lentiviral hematopoietic stem cell gene therapy in patients with Wiskott-Aldrich syndrome. Science 341: 1233151 10.1126/science.1233151 23845947 PMC4375961

[32] Su IH, Dobenecker MW, Dickinson E, Oser M, Basavaraj A, et al. (2005) Polycomb group protein ezh2 controls actin polymerization and cell signaling. Cell 121: 425–436. 10.1016/j.cell.2005.02.029 15882624

[33] Tong ZT, Cai MY, Wang XG, Kong LL, Mai SJ, et al. (2012) EZH2 supports nasopharyngeal carcinoma cell aggressiveness by forming a co-repressor complex with HDAC1/HDAC2 and Snail to inhibit E-cadherin. Oncogene 31: 583–594. 10.1038/onc.2011.254 21685935

[34] Kottakis F, Polytarchou C, Foltopoulou P, Sanidas I, Kampranis SC, et al. (2011) FGF-2 regulates cell proliferation, migration, and angiogenesis through an NDY1/KDM2B-miR-101-EZH2 pathway. Mol Cell 43: 285–298. 10.1016/j.molcel.2011.06.020 21777817 PMC3324394

[35] Tang TC, Poon RT, Lau CP, Xie D, Fan ST (2005) Tumor cyclooxygenase-2 levels correlate with tumor invasiveness in human hepatocellular carcinoma. World J Gastroenterol 11: 1896–1902. 15800977 10.3748/wjg.v11.i13.1896 PMC4305708

[36] Leng J, Han C, Demetris AJ, Michalopoulos GK, Wu T (2003) Cyclooxygenase-2 promotes hepatocellular carcinoma cell growth through Akt activation: evidence for Akt inhibition in celecoxib-induced apoptosis. Hepatology 38: 756–768. 10.1053/jhep.2003.50380 12939602

[37] Reichmann E, Schwarz H, Deiner EM, Leitner I, Eilers M, et al. (1992) Activation of an inducible c-FosER fusion protein causes loss of epithelial polarity and triggers epithelial-fibroblastoid cell conversion. Cell 71: 1103–1116. 10.1016/S0092-8674(05)80060-1 1473147

[38] Yuan RH, Jeng YM, Chen HL, Lai PL, Pan HW, et al. (2006) Stathmin overexpression cooperates with p53 mutation and osteopontin overexpression, and is associated with tumour progression, early recurrence, and poor prognosis in hepatocellular carcinoma. J Pathol 209: 549–558. 10.1002/path.2011 16739096

[39] Chen Y, Lin MC, Yao H, Wang H, Zhang AQ, et al. (2007) Lentivirus-mediated RNA interference targeting enhancer of zeste homolog 2 inhibits hepatocellular carcinoma growth through down-regulation of stathmin. Hepatology 46: 200–208. 10.1002/hep.21668 17596871

[40] Wong CC, Wong CM, Tung EK, Man K, Ng IO (2009) Rho-kinase 2 is frequently overexpressed in hepatocellular carcinoma and involved in tumor invasion. Hepatology 49: 1583–1594. 10.1002/hep.22836 19205033

[41] He QW, Xia YP, Chen SC, Wang Y, Huang M, et al. (2013) Astrocyte-derived sonic hedgehog contributes to angiogenesis in brain microvascular endothelial cells via RhoA/ROCK pathway after oxygen-glucose deprivation. Mol Neurobiol 47: 976–987. 10.1007/s12035-013-8396-8 23325464

[42] Zhang Z, Yang M, Chen R, Su W, Li P, et al. (2013) IBP regulates epithelial-to-mesenchymal transition and the motility of breast cancer cells via Rac1, RhoA and Cdc42 signaling pathways. Oncogene 33(26):3374–3382. 23975422 10.1038/onc.2013.337 PMC4078416

[43] Chen L, Chan TH, Yuan YF, Hu L, Huang J, et al. (2010) CHD1L promotes hepatocellular carcinoma progression and metastasis in mice and is associated with these processes in human patients. J Clin Invest 120: 1178–1191. 10.1172/JCI40665 20335658 PMC2846051

[44] Xu N, Bagumian G, Galiano M, Myat MM (2011) Rho GTPase controls Drosophila salivary gland lumen size through regulation of the actin cytoskeleton and Moesin. Development 138: 5415–5427. 10.1242/dev.069831 22071107 PMC3222215

[45] Mitchell PS, Parkin RK, Kroh EM, Fritz BR, Wyman SK, et al. (2008) Circulating microRNAs as stable blood-based markers for cancer detection. Proc Natl Acad Sci U S A 105: 10513–10518. 10.1073/pnas.0804549105 18663219 PMC2492472

[46] Huang Z, Huang D, Ni S, Peng Z, Sheng W, et al. (2010) Plasma microRNAs are promising novel biomarkers for early detection of colorectal cancer. Int J Cancer 127: 118–126. 10.1002/ijc.25007 19876917

[47] Zhu W, Cai MY, Tong ZT, Dong SS, Mai SJ, et al. (2012) Overexpression of EIF5A2 promotes colorectal carcinoma cell aggressiveness by upregulating MTA1 through C-myc to induce epithelial-mesenchymaltransition. Gut 61: 562–575. 10.1136/gutjnl-2011-300207 21813470

