# Correction: Systemic Delivery of MicroRNA-101 Potently Inhibits Hepatocellular Carcinoma *In Vivo* by Repressing Multiple Targets

**DOI:** 10.1371/journal.pgen.1009960

**Published:** 2021-12-08

**Authors:** Fang Zheng, Yi-Ji Liao, Mu-Yan Cai, Tian-Hao Liu, Shu-Peng Chen, Pei-Hong Wu, Long Wu, Xiu-Wu Bian, Xin-Yuan Guan, Yi-Xin Zeng, Yun-Fei Yuan, Hsiang-Fu Kung, Dan Xie

Fig 3, Fig 4, S3 Fig, S6 Fig, and S8 Fig are incorrect. There are errors in panels C, D and E of Fig 3, panel C of Fig 4, panels C and D of S3 Fig, panel A of S6 Fig, and panel A of S8 Fig. The authors have provided corrected versions below.

**Fig 3:**
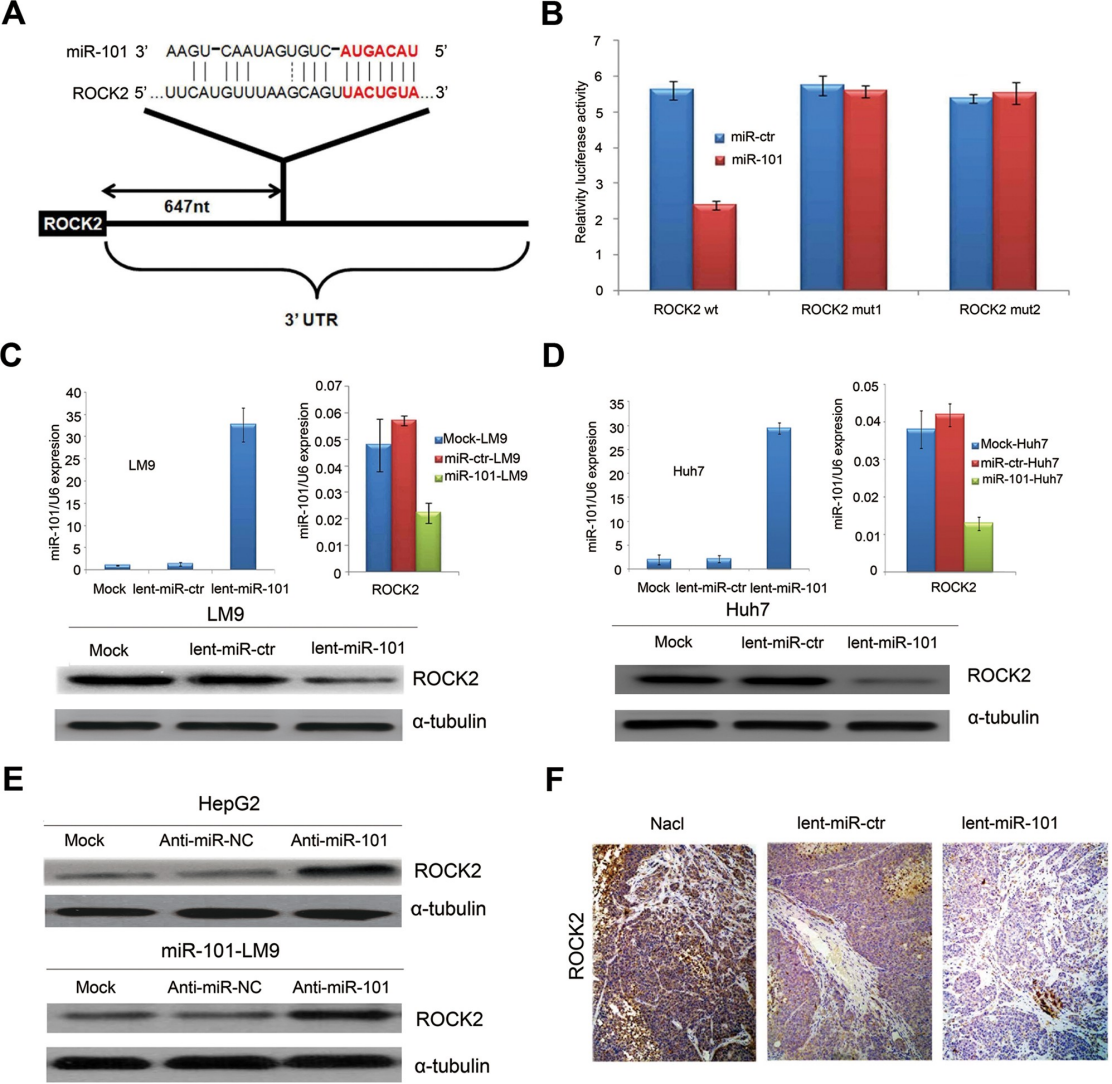
*ROCK2* is the target of miR-101. (A) Schematic of predicted miR-101-binding sites in the 3′UTR of *ROCK2*. (B) MiR report constructs containing a wild-type and 2 mutated ROCK2 3’UTR were transfected into LM9 cells, respectively. Relative repression of firefly luciferase expression was standardized to a transfection control. The reporter assays were performed 3 times with essentially identical results. (C) Left, the levels of miR-101 by Real-time PCR in the lenti-miR-101 and control MOCK and lent-miR-ctr treated LM9 cells. Right, real-time PCR examination of mRNA levels of *ROCK2* between the lenti-miR-101 and control lent-miR-ctr treated LM9 cells. LM9 cells were infected with lent-miR-ctr or lent-miR-101 for 72 hours. Down, ectopic overexpression of miR-101 by lenti-miR-101 reduces the levels of *ROCK2* proteins in LM9 cells, as compared to that in both MOCK and lent-miR-ctr treated LM9 cells. (D) Left, the levels of miR-101 by Real-time PCR in the lenti-miR-101 and mock and lent-miR-ctr treated Huh7 cells. Right, real-time PCR examination of mRNA level of *ROCK2* between the lenti-miR-101 and control lent-miR-ctr treated Huh7 cells. Huh7 cells were infected with lent-miR-ctr or lent-miR-101 for 72 hours. Down, ectopic overexpression of miR-101 by lenti-miR-101 reduces the levels of *ROCK2* proteins in Huh7 cells, as compared to that in both Mock and lent-miR-ctr treated Huh7 cells. (E) Upper, protein expression of *ROCK2* is up-regulated in HCC HepG2 cells after the down-regulation of miR-101 by anti-miR-101, as compared to that in control Mock and anti-miR-NC HepG2 cells. Down, protein expressions of *ROCK2* is up-regulated in lent-miR-101-LM9 cells after the down-regulation of miR-101 by anti-miR-101, as compared to that in control anti-miR-NC cells. (F) IHC staining showing down-regulated expressions of ROCK2 in HCC tissues of mice treated with systemic delivery of lent-miR-101, as compared to that treated with NaCl or lent-miR-ctr.

**Fig 4:**
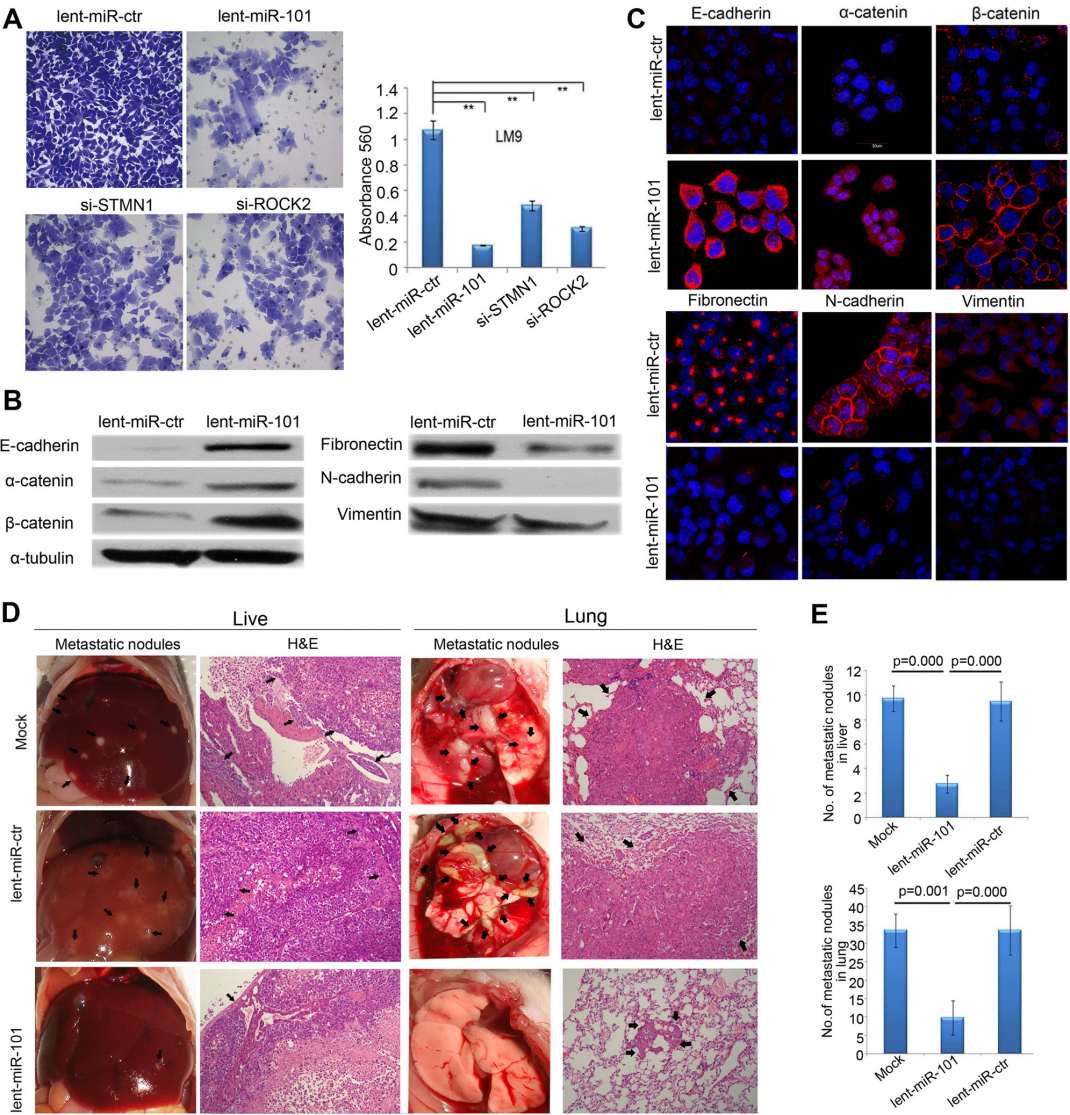
Enforced overexpression of miR-101 inhibits HCC LM9 cells invasion and EMT *in vitro* and reduces metastasis *in vivo*. (A) The invasive properties of HCC LM9 cells transfected with lent-miR-ctr, lent-miR-101, si-S*TMN1*, and si-*ROCK2* were analyzed by an invasion assay using a Matrigel^TM^ Invasion Chamber. Migrated cells were plotted as the average number of cells per field of view from 3 independent experiments (**, *P*<0.01). (B) Expression levels of the epithelial markers E-cadherin, α-catenin and β-catenin and the mesenchymal markers fibronectin, N-cadherin and vimentin were analyzed by Western blot between lent-miR-101 and control lent-miR-ctr LM9 cells. (C) IF staining (red signal) showing that the Epithelial markers E-cadherin, α-catenin, β-catenin were up-regulated and mesenchymal markers fibronectin, N-cadherin and vimentin were down-regulated in lent-miR-101 treated LM9 cells, as compare to that in lent-miR-ctr cells. (D) The *in vivo* effects of miR-101 on HCC cell metastasis using an experimental metastasis assay, in which lent-miR-101, control lent-miR-ctr and mock LM9 cells were injected into the tail vein of SCID mice, respectively. Metastatic tumor growth in the liver and in the lung was assessed. Representative metastatic nodules and H&E staining of metastatic tumors in the liver and in the lung are indicated by arrows. **(**E) The number of metastatic nodules in the liver and in the lungs of mice (*n* = 8 per group) 8 weeks after tail vein injection of let-miR-101 LM9 cells (mean±SE, liver: 2.8±0.8, lung: 9.8±4.7), mock LM9 cells (mean±SE, liver: 9.8±1.0, lung: 33.5±4.6) and lent-miR-ctr LM9 cells (mean±SE, liver: 9.5± 1.6, lung: 33.5± 6.7).

The following information is missing from the Methods section: the Western blots for STMN1 and α-tubulin in S3 Fig were from independent gel runs.

## Supporting information

S3 Fig *STMN1* is the target of miR-101. (A) Schematic of predicted miR-101-binding sites in the 3′UTR of *STMN1*. (B) MiR report constructs containing a wild-type and 2 mutated *STMN1* 3’UTRs were transfected into LM9 cells, respectively. Relative repression of firefly luciferase expression was standardized to a transfection control. The reporter assays were performed 3 times with essentially identical results. (C) Upper, real-time PCR examination of mRNA levels of *ROCK2* between the lenti-miR-101 and control lent-miR-ctr treated LM9 cells. LM9 cells were infected with lent-miR-ctr or lent-miR-101 for 72 hours. Down, ectopic overexpression of miR-101 by lenti-miR-101 reduces the levels of *STMN1* protein in LM9 cells, as compared to that in both Mock and lent-miR-ctr treated LM9 cells. (D) Protein expression of *STMN1* is up-regulated in HCC HepG2 cells after the down-regulation of miR-101 by anti-miR-101, as compared to that in control Mock and anti-miR-NC HepG2 cells. (E) IHC staining showing down-regulated expressions of *STMN1* in HCC tissues of mice treated with systemic delivery of lent-miR-101, as compared to that treated with NaCl or lent-miR-ctr.
(TIF)

S6 Fig Ectopic overexpression of miR-101 inhibits HCC Huh7 cell invasion and EMT *in vitro*. (A) The invasive properties of HCC Huh cells transfected with lent-miR-ctr, lent-miR-101, si-*STMN1*, and si-*ROCK2* were analyzed by an invasion assay using a Matrigel^TM^ Invasion Chamber. Migrated cells were plotted as the average number of cells per field of view from 3 indipendent experiments (**, *P*<0.01). (B) Expression levels of the epithelial markers E-cadherin, α-catenin, β-catenin and the mesenchymal markers fibronectin, N-cadherin and vimentin were analyzed by Western blot between lent-miR-101 and control lent-miR-ctr treated Huh cells. (C) IF staining was used to compare expression levels/pattern of epithelial markers and mesenchymal markers (red signal) between the control lent-miR-ctr and lent-miR-101 treated Huh cells. The Epithelial markers E-cadherin, α-catenin, β-catenin were upregulated and mesenchymal markers fibronectin, N-cadherin and vimentin were downregulated in lent-miR-101 treated Huh cells, as compare to that in lent-miR-ctr Huh cells.
(TIF)

S8 Fig Enforced expression of miR-101 in HCC cell line inhibits the mRNA and protein levels of *EZH2*. (A) Enforced overexpression of miR-101 in LM9 cells decreases endogenous levels of *EZH2* protein. LM9 cells were infected with Mock, lent-miR-ctr or lenti-miR-101 for 72 hours. *EZH2* expression was assessed by Western blot. (B) The mRNA levels of *EZH2* in Mock, lent-miR-ctr or lenti-miR-101 LM9 cells examined by Real-time PCR. Lenti-miR-101 decreased the levels of *EZH2* mRNA in LM9 cells. (C) Western blot assay showing protein levels of *EZH2* after the treatment of Mock, Anti-miRNC and anti-miR-101 in HepG2 cell line. Anti-miR-101 could increase *EZH2* expression in HepG2 cells.
(TIF)
