# Supplementary figures and images for: Systemic Delivery of MicroRNA-101 Potently Inhibits Hepatocellular Carcinoma In Vivo by Repressing Multiple Targets
Source: PLoS Genet. 2015 Feb 18;11(2):e1004873. doi: 10.1371/journal.pgen.1004873 (PMC4334495; doi:10.1371/journal.pgen.1004873)

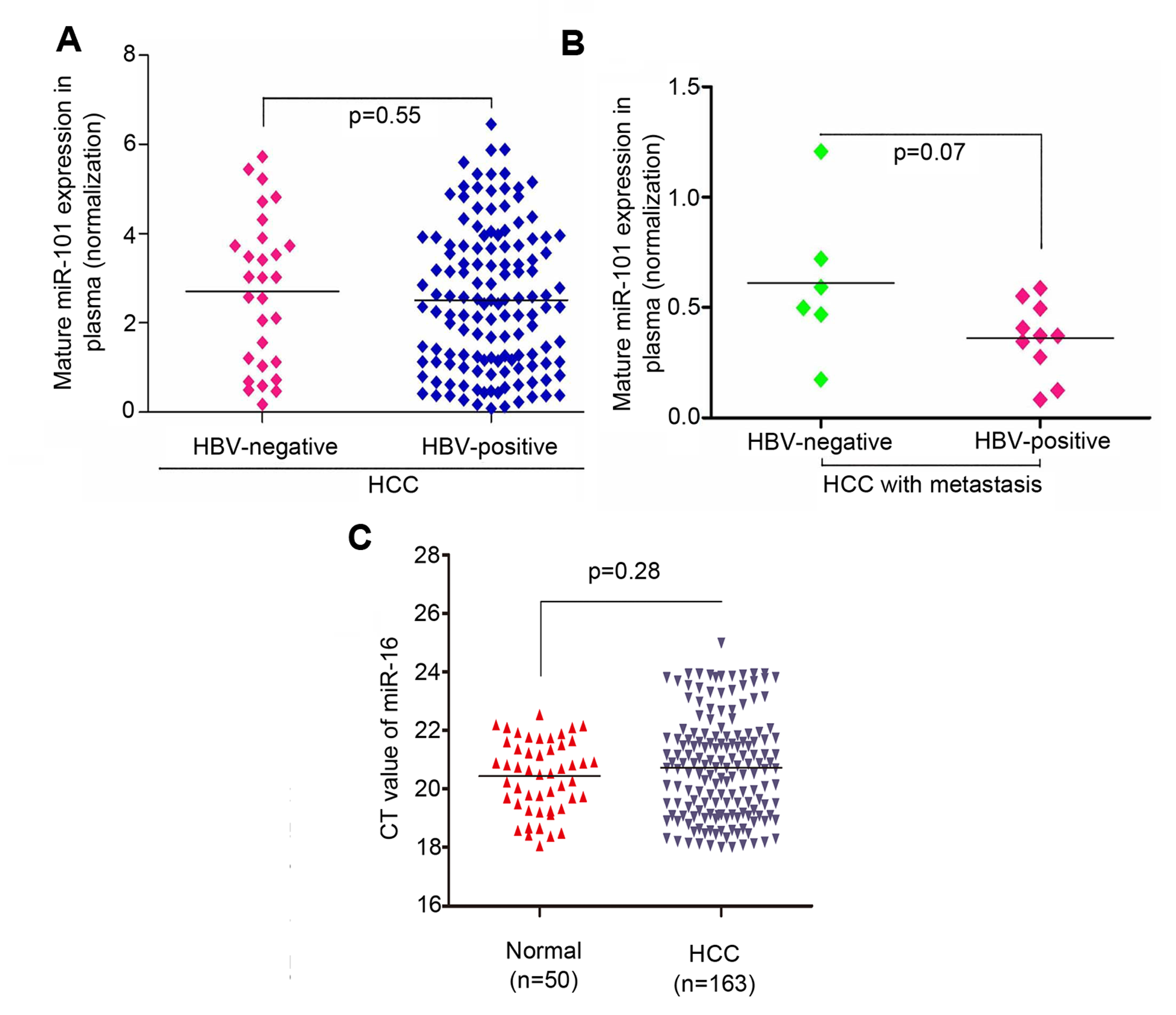

Supplement: S1 Fig — A. The level of mature miR-101 in HBV-negative (n = 29) and HBV-positive (n = 134) HCC patient’s plasma. B. The level of mature miR-101 in HBV-negative (n = 5) and HBV-positive (n = 10) HCC patient’s plasma with distant metastasis. C. CT value of miR-16 in human plasma samples from healthy donors (normal, n = 50) and HCC patients (n = 163). (TIF) [file pgen.1004873.s001.tif]

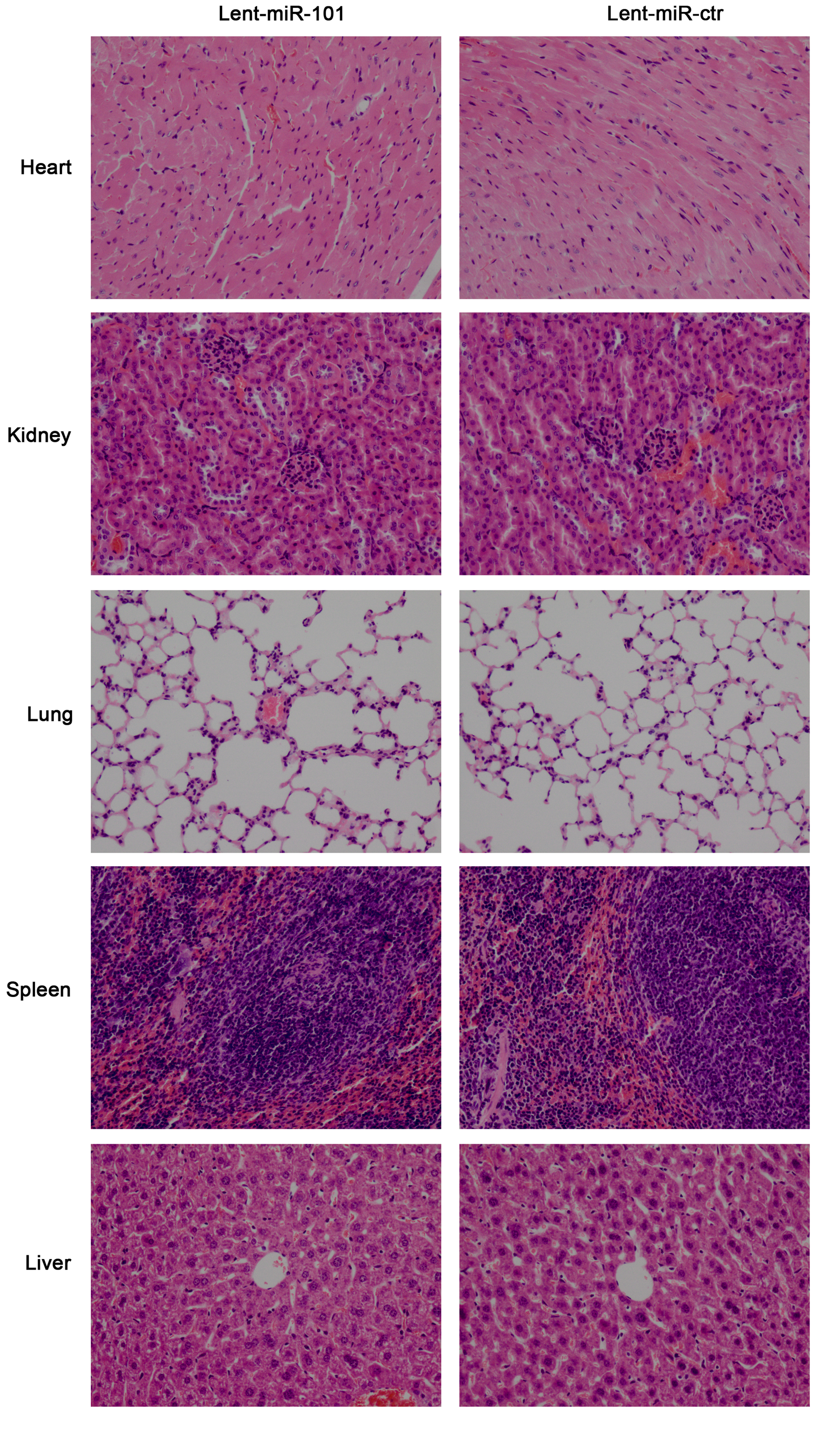

Supplement: S2 Fig — Lent-miR-101 and control lent-miR-ctr was administered, respectively, to mice by tail vein at one week after the preparation of the mouse HCC model, 2 times a week for a month. After the experiment, animals were sacrificed and the organs were fixed in formalin overnight and processed for paraffin embedding. The paraffin-embedded blocks were sectioned and stained by hematoxylin and eosin. (TIF) [file pgen.1004873.s002.tif]

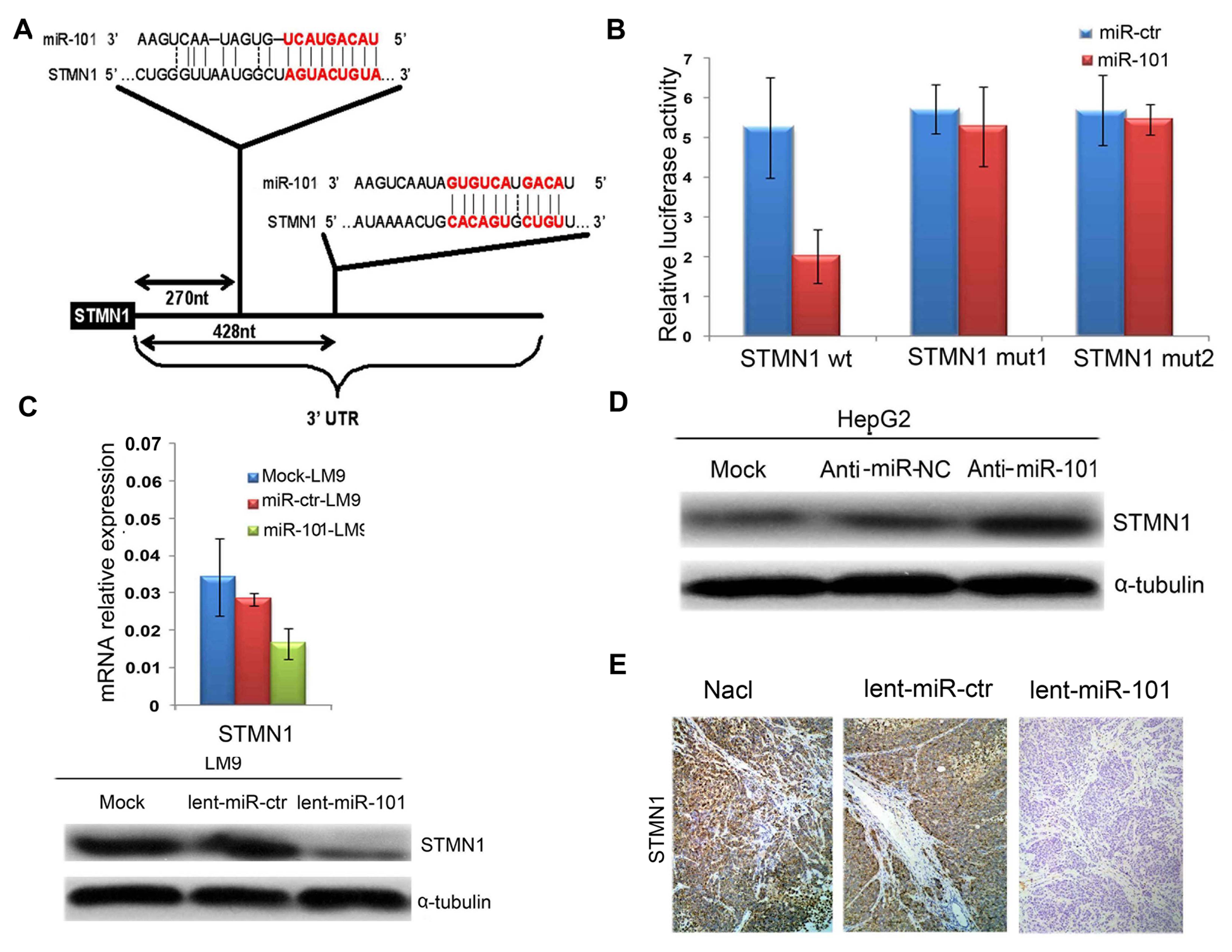

Supplement: S3 Fig — A. Schematic of predicted miR-101-binding sites in the 3′UTR of STMN1. B. MiR report constructs containing a wild-type and 2 mutated STMN1 3’UTRs were transfected into LM9 cells, respectively. Relative repression of firefly luciferase expression was standardized to a transfection control. The reporter assays were performed 3 times with essentially identical results. C. Upper, real-time PCR examination of mRNA levels of ROCK2 between the lenti-miR-101 and control lent-miR-ctr treated LM9 cells. LM9 cells were infected with lent-miR-ctr or lent-miR-101 for 72 hours. Down, ectopic overexpression of miR-101 by lenti-miR-101 reduces the levels of STMN1 protein in LM9 cells, as compared to that in both Mock and lent-miR-ctr treated LM9 cells. D. Protein expression of STMN1 is up-regulated in HCC HepG2 cells after the down-regulation of miR-101 by anti-miR-101, as compared to that in control Mock and anti-miR-NC HepG2 cells. E. IHC staining showing down-regulated expressions of STMN1 in HCC tissues of mice treated with systemic delivery of lent-miR-101, as compared to that treated with NaCl or lent-miR-ctr. (TIF) [file pgen.1004873.s003.tif]

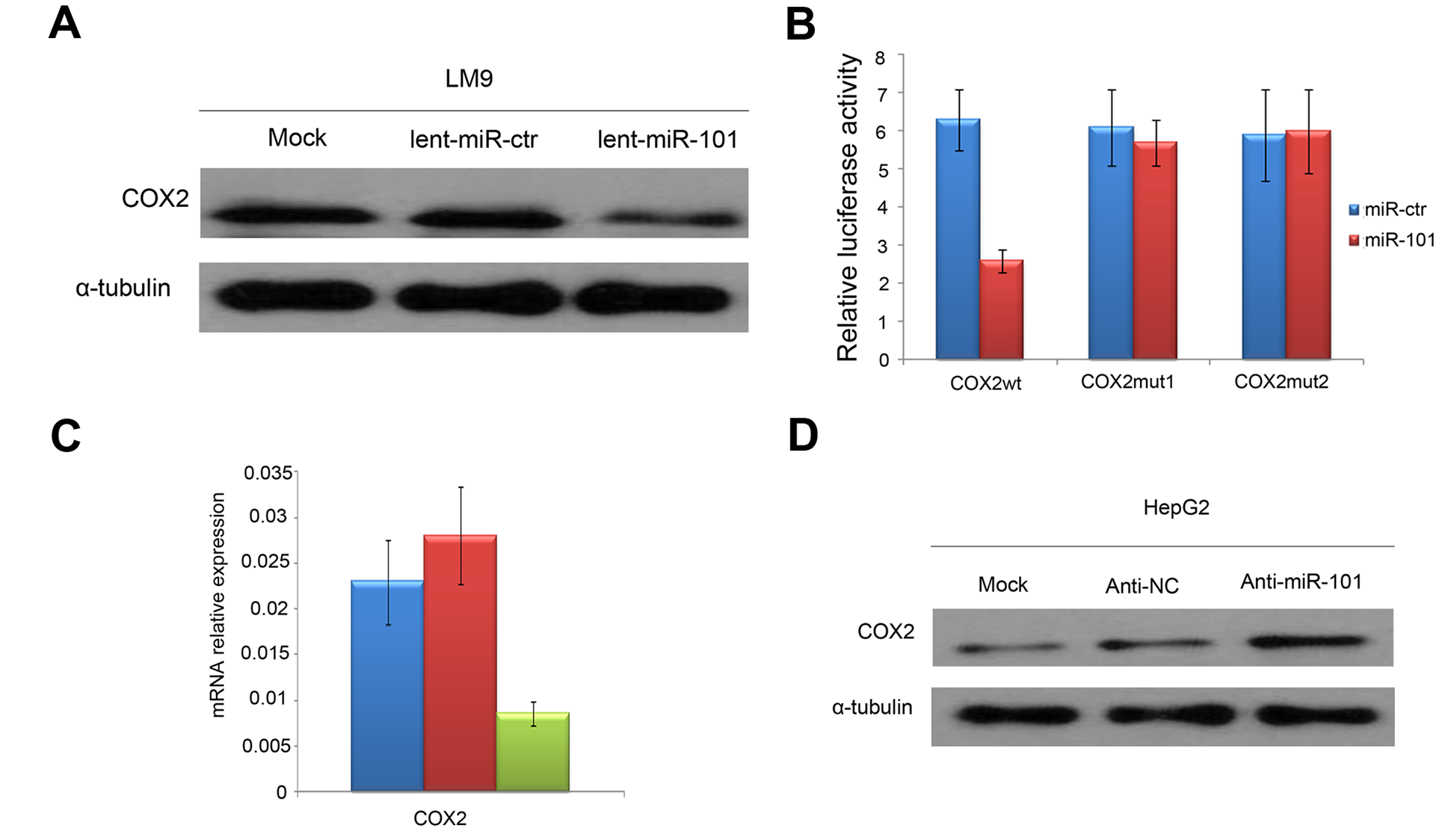

Supplement: S4 Fig — A. Enforced overexpression of miR-101 in LM9 cells decreases endogenous levels of COX2 protein. LM9 cells were infected with Mock, lent-miR-ctr or lenti-miR-101 for 72 hours. COX2 expression was assessed by Western blot. B. MiR report constructs containing a wild-type and 2 mutated COX2 3’UTRs were transfected into LM9 cells, respectively. Relative repression of firefly luciferase expression was standardized to a transfection control. The reporter assays were performed 3 times with essentially identical results. C. The mRNA levels of COX2 in Mock, lent-miR-ctr or lenti-miR-101 LM9 cells examined by Real-time PCR. Lenti-miR-101 decreased the levels of COX2 mRNA in LM9 cells. D. Western blot assay showing protein levels of COX2 after the treatment of Mock, Anti-miRNC and anti-miR-101 in HepG2 cell line. Anti-miR-101 could increase COX2 expression in HepG2 cells. (TIF) [file pgen.1004873.s004.tif]

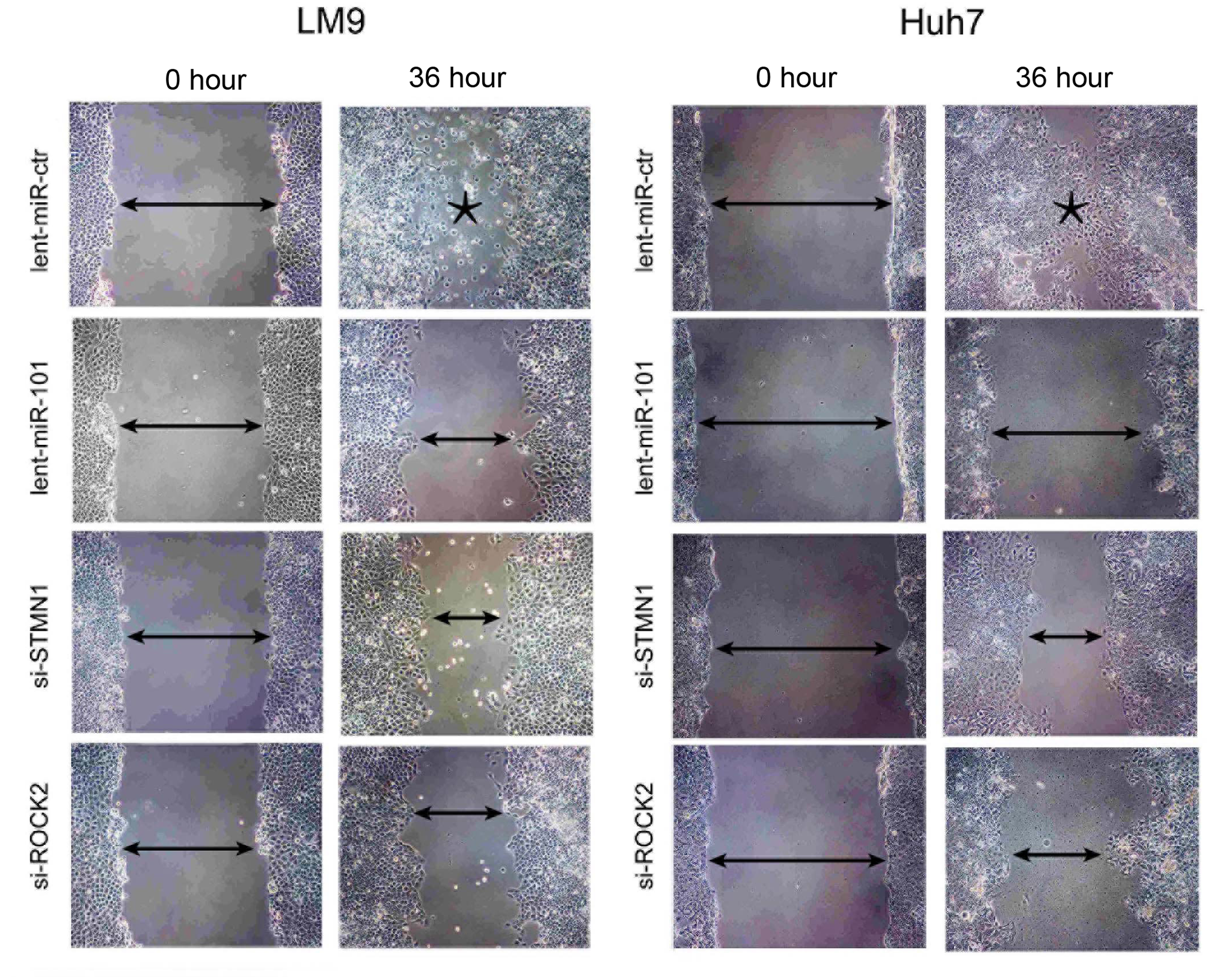

Supplement: S5 Fig — Ectopic overexpression of miR-101 by infection of lent-miR-101 decreased cell motility in both LM9 and Huh7 cells, compared with that in lent-miR-ctr control cells. Silence of either ROCK2 or STMN1 by specific siRNA could partially mimic the inhibiting effect of lent-miR-101 on both HCC cells motilities. (TIF) [file pgen.1004873.s005.tif]

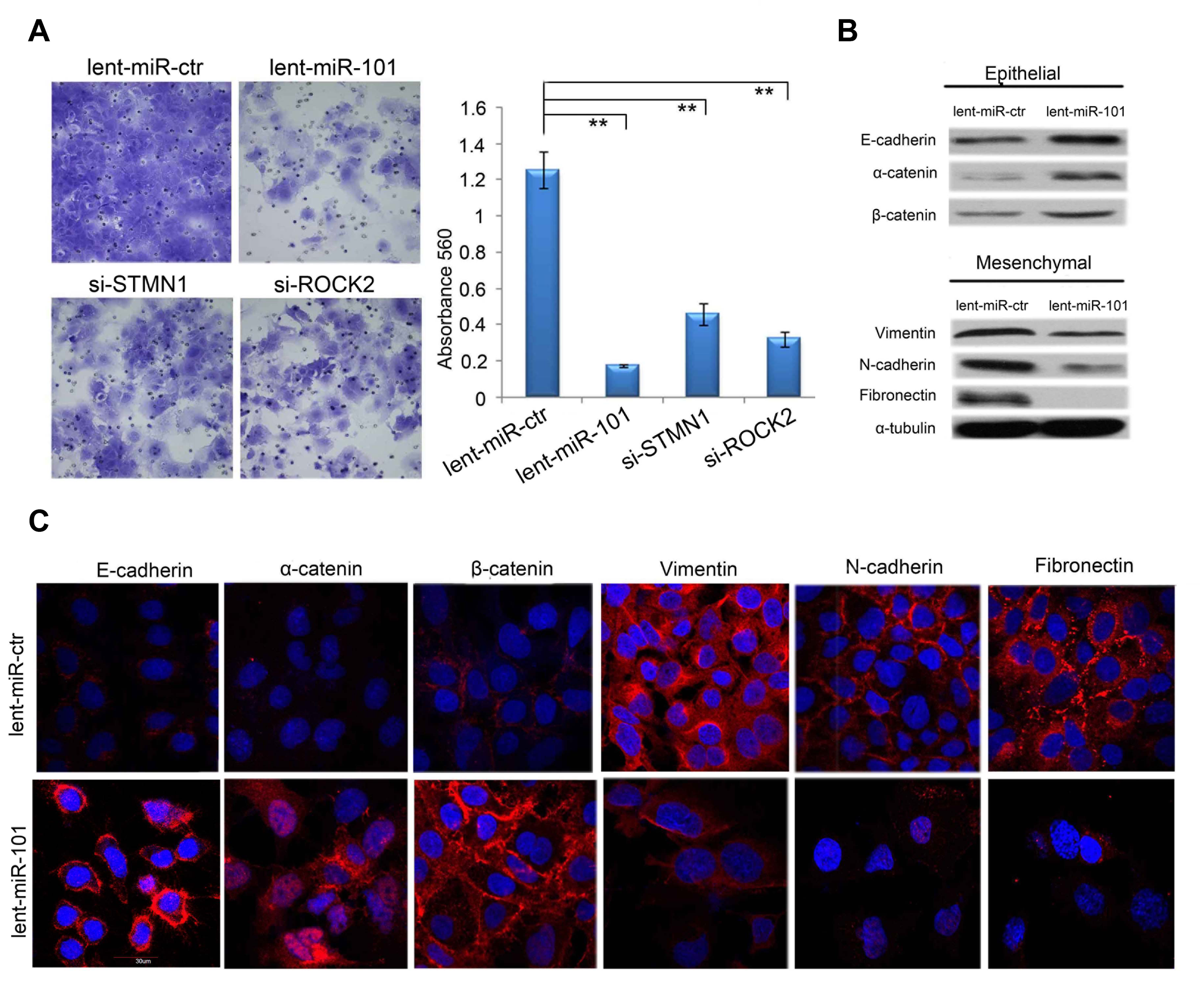

Supplement: S6 Fig — A. The invasive properties of HCC Huh cells transfected with lent-miR-ctr, lent-miR-101, si-STMN1, and si-ROCK2 were analyzed by an invasion assay using a Matrigel Invasion Chamber. Migrated cells were plotted as the average number of cells per field of view from 3 indipendent experiments (**, P<0.01). B. Expression levels of the epithelial markers E-cadherin, α-catenin, β-catenin and the mesenchymal markers fibronectin, N-cadherin and vimentin were analyzed by Western blot between lent-miR-101 and control lent-miR-ctr treated Huh cells. C. IF staining was used to compare expression levels/pattern of epithelial markers and mesenchymal markers (red signal) between the control lent-miR-ctr and lent-miR-101 treated Huh cells. The Epithelial markers E-cadherin, α-catenin, β-catenin were upregulated and mesenchymal markers fibronectin, N-cadherin and vimentin were downregulated in lent-miR-101 treated Huh cells, as compare to that in lent-miR-ctr Huh cells. (TIF) [file pgen.1004873.s006.tif]

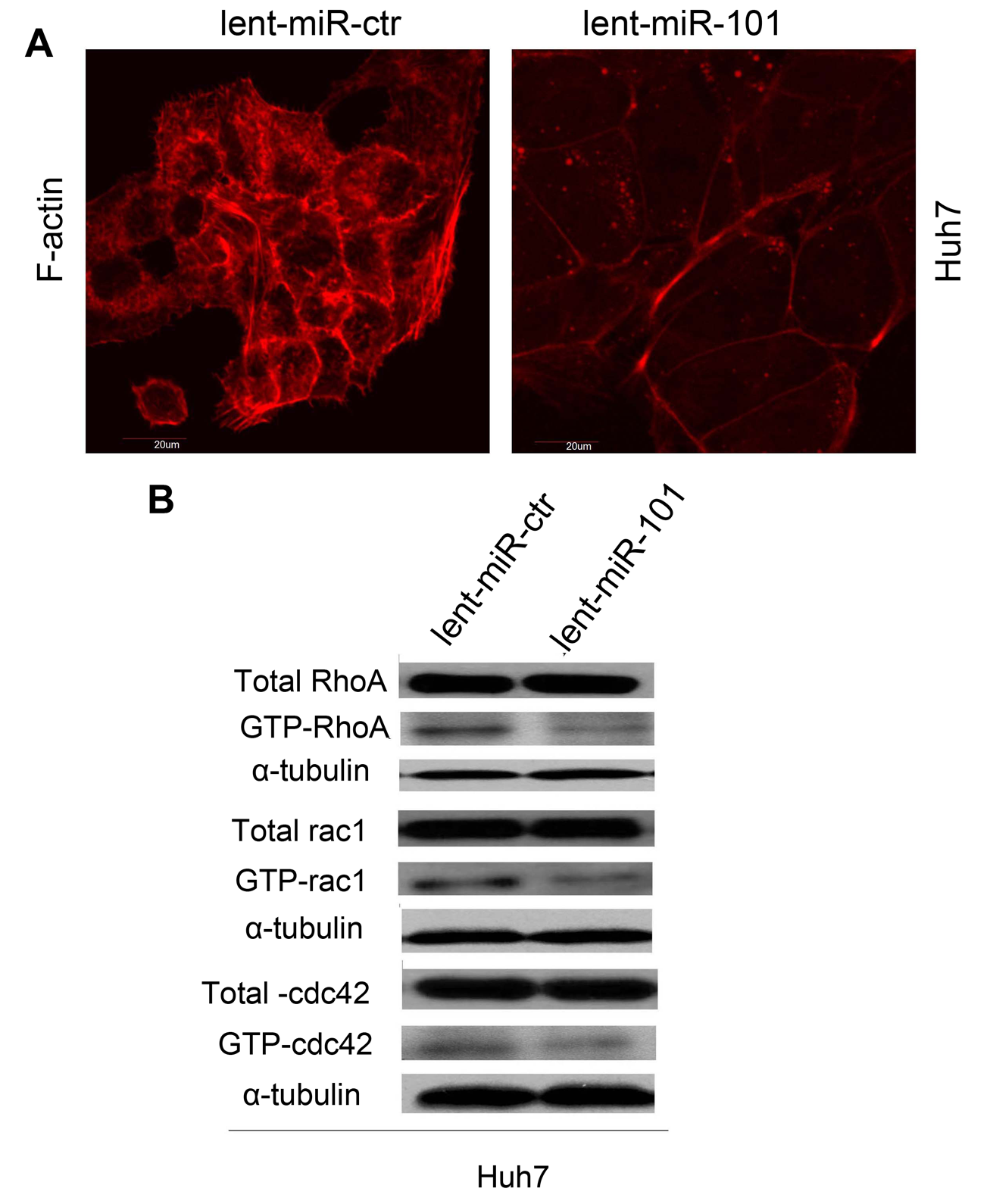

Supplement: S7 Fig — A. Staining for F-actin demonstrated that the stress fiber formation in lent-miR-101 treated Huh7 cells decreased when compared with that in lent-miR-ctr Huh7 cells. B. Total and active forms of Rho-GTPases, including RhoA, Rac1, and cdc42 were compared between lent-miR-ctr and lent-miR-101 treated Huh7 cells by Western blot analysis. GTP-bound (active) forms of RhoA, Rac1, and cdc42 were pulled down and examined by Western blot using corresponding antibodies. Active forms of RhoA, Rac1 and Cdc42 were lower in lent-miR-101 Huh7 than that in lent-miR-ctr Huh7 cells. (TIF) [file pgen.1004873.s007.tif]

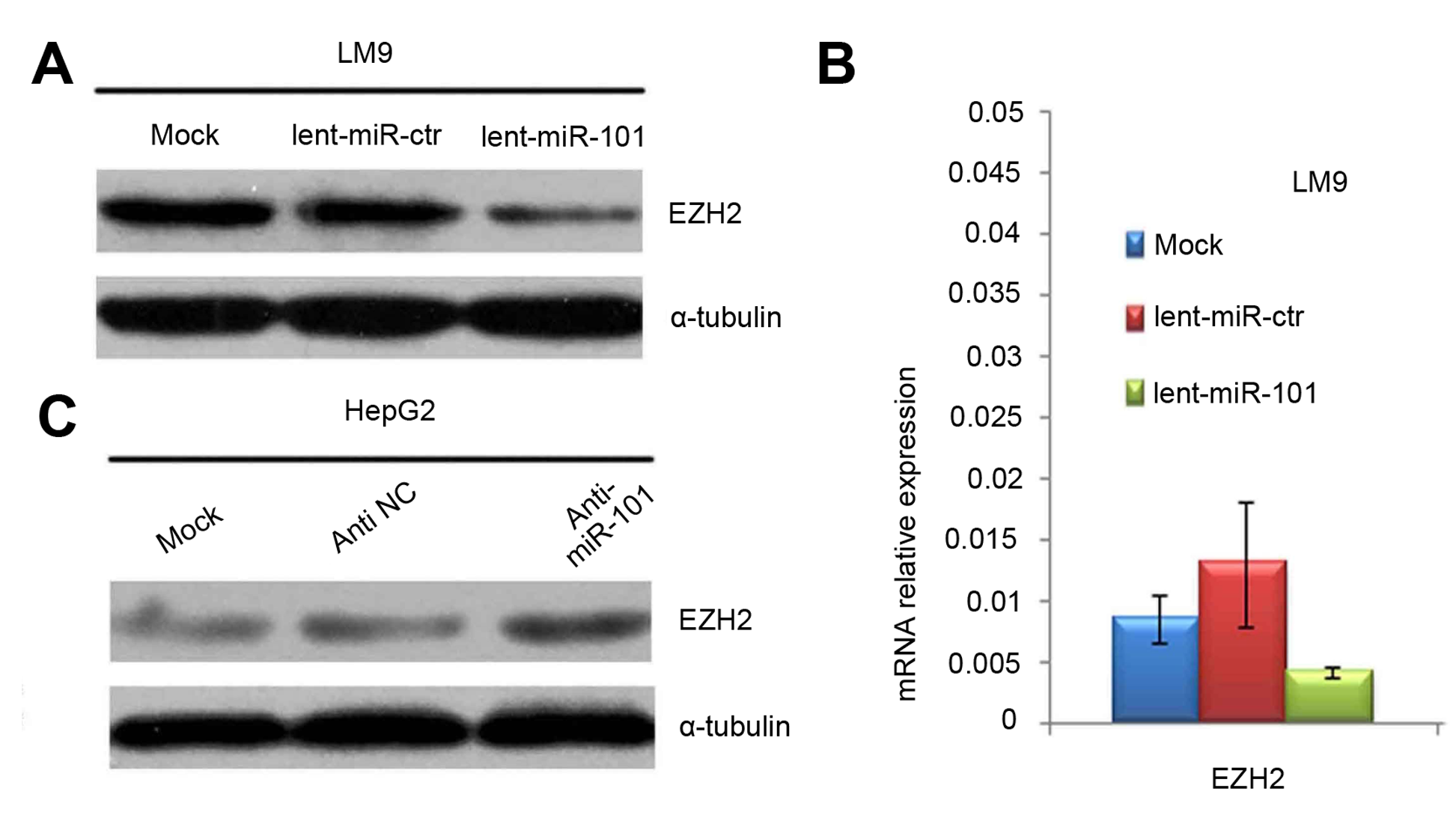

Supplement: S8 Fig — A. Enforced overexpression of miR-101 in LM9 cells decreases endogenous levels of EZH2 protein. LM9 cells were infected with Mock, lent-miR-ctr or lenti-miR-101 for 72 hours. EZH2 expression was assessed by Western blot. B. The mRNA levels of EZH2 in Mock, lent-miR-ctr or lenti-miR-101 LM9 cells examined by Real-time PCR. Lenti-miR-101 decreased the levels of EZH2 mRNA in LM9 cells. C. Western blot assay showing protein levels of EZH2 after the treatment of Mock, Anti-miRNC and anti-miR-101 in HepG2 cell line. Anti-miR-101 could increase EZH2 expression in HepG2 cells. (TIF) [file pgen.1004873.s008.tif]
