# Supplementary material for: Systemic Delivery of MicroRNA-101 Potently Inhibits Hepatocellular Carcinoma In Vivo by Repressing Multiple Targets
Source: PLoS Genet. 2015 Feb 18;11(2):e1004873. doi: 10.1371/journal.pgen.1004873 (PMC4334495; doi:10.1371/journal.pgen.1004873)
Supplement: S1 Table — Main serological parameters of 3 groups of nude mice at the study endpoints. (DOC) [file pgen.1004873.s009.doc]

**Table S1.** Main serological parameters of 3 groups of nude mice at the study endpoints

| | **Biochemical parameters of blood** | | | | | | | | | | --- | --- | --- | --- | --- | --- | --- | --- | --- | |  | **TB** | **DB** | **ALT** | **AST** | **TP** | **ALB** | **A/G** | **AFP** | | SG | 1.8±0.237 | 0.19±0.074 | 95.5±36.5 | 97.9±33.8 | 54.2±3.99 | 30.7±2.36 | 1.34±0.24 | 643.2±126.5 | | VG | 1.7±0.256 | 0.18±0.114 | 88.1±20.4 | 91.1±23.1 | 55.7±3.34 | 32.6±2.72 | 1.43±0.25 | 626.4±130.4 | | TG | 1.6±0.234 | 0.19±0.053 | 91.1±23.6 | 92.3±16.1 | 63.6±4.35 | 36.7±2.69 | 1.62±0.21 | 276.7±56.9 | |
| --- | --- | --- | --- | --- | --- | --- | --- | --- | --- | --- | --- | --- | --- | --- | --- | --- | --- | --- | --- | --- | --- | --- | --- | --- | --- | --- | --- | --- | --- | --- | --- | --- | --- | --- | --- | --- | --- | --- | --- | --- | --- | --- | --- | --- | --- |

SG = saline group; VG = vector group; TG = treatment group; TB = total bilirubin (μmol/l); DB = direct bilirubin (μmol/l); ALT = alanine aminotransferase (U/I); AST = aspartate aminotransferase (U/I); TP = total protein (g/l); ALB = albumin (g/l); A/G = albumin/globulin ratio; AFP = alpha fetoprotein (μg/l).
