# Supplementary material for: Systemic Delivery of MicroRNA-101 Potently Inhibits Hepatocellular Carcinoma In Vivo by Repressing Multiple Targets
Source: PLoS Genet. 2015 Feb 18;11(2):e1004873. doi: 10.1371/journal.pgen.1004873 (PMC4334495; doi:10.1371/journal.pgen.1004873)
Supplement: S2 Table — Primers for ROCK2, STMN1 and COX2 3’-UTRs in the luciferase report assay and real-time PCR primers for ROCK2, STMN1, EZH2 and COX2. (DOC) [file pgen.1004873.s010.doc]

**Table S2.** Primers for ROCK2, STMN1 and COX2 3’-UTRs in the luciferase report assay and real-time PCR primers for ROCK2, STMN1, EZH2 and COX2

| **Primers** | **Sequences** |
| --- | --- |
| m101-ROCK2-F1 | GGACTAGTGGATTCCACTACAGTGATATTG |
| m101-ROCK2-R1 | CCCAAGCTTGTGCACGATTTATCTTCCAC |
| m101-ROCK2-F1MDR | ATTAACTTTAAACTTCTTGT**A**CTGCTTAAACATGAACATG |
| m101-ROCK2-R1MDF | CAGTACAAGAAGTTTAAAGTTAAT |
| m101-ROCK2-F1MSR | ATTAACTTTAAACTTCTTGTATGTCATACTGCTTAAACATGAACATG |
| m101-ROCK2-R1MSF | TATGACATACAAGAAGTTTAAAGTTAAT |
| m101-STMN1-F2 | GGACTAGTTTGTTCTGAGAACTGACTT |
| m101-STMN1-R2 | CCCAAGCTTGCTACAGCAGTACATAAAG |
| m101-STMN1-R2MDF | ACTGTGGTGGCAGTGACTTCTTTT |
| m101-STMN1-F2MDR | ATATGTTTTCACAGAGCCAAAGCCATTAACCCAGTACACC |
| m101-STMN1-MDF1 | GGCTTTGGCTCTGTGAAAACATAT |
| m101-STMN1-MDR1 | AAAAGAAGTCACTGCCACCACAGTTTTATTAACCATTCAA |
| m101-STMN1-R2MSF | GGTGTCAGGACATGGTGGCAGTGACTTCTTTT |
| m101-STMN1-F2MSR | ATATGTTTTCACAGAGCCAAATGTCATGAAGCCATTAACCCAGTACACC |
| m101-STMN1-MSF1 | TTCATGACATTTGGCTCTGTGAAAACATAT |
| m101-STMN1-MSR1 | AAAAGAAGTCACTGCCACCATGTCCTGACACCAGTTTTATTAACCATTCAA |
| m101-COX2-F1 | GGACTAGTGCTATCTGTAACCAAGATGG |
| m101- COX2-F1MDR | TTCAATAAATTAAGTAATATCATTAAATGTCAGTGACAAT |
| m101- COX2-R1MDF | AATGATATTACTTAATTTATTGAA |
| m101- COX2-R1 | CCCAAGCTTCACATAGGCCTATCCTAAGG |
| ROCK2-qPF | ATGAAGATACAGCAAAACCAGTC |
| ROCK2-qPR | CACCTTGAATAATGACTGCTTTC |
| STMN1-qPF | CTCGGACTGAGCAGGACTTTC |
| STMN2-qPR | GCACGCTTCTCCAGTTCTTTC |
| EZH2-qPF | TGCAGTTGCTTCAGTACCCATAAT |
| EZH2-qPR | ATCCCCGTGTACTTTCCCATCATAAT |
| COX2-qPF | TCTGGCTAGACAGCGTAA |
| COX2-qPR | TCAGGGACTTGAGGAGGG |
