# Supplementary figures and images for: Correction: Systemic Delivery of MicroRNA-101 Potently Inhibits Hepatocellular Carcinoma In Vivo by Repressing Multiple Targets
Source: PLoS Genet. 2021 Dec 8;17(12):e1009960. doi: 10.1371/journal.pgen.1009960 (PMC8654154; doi:10.1371/journal.pgen.1009960)

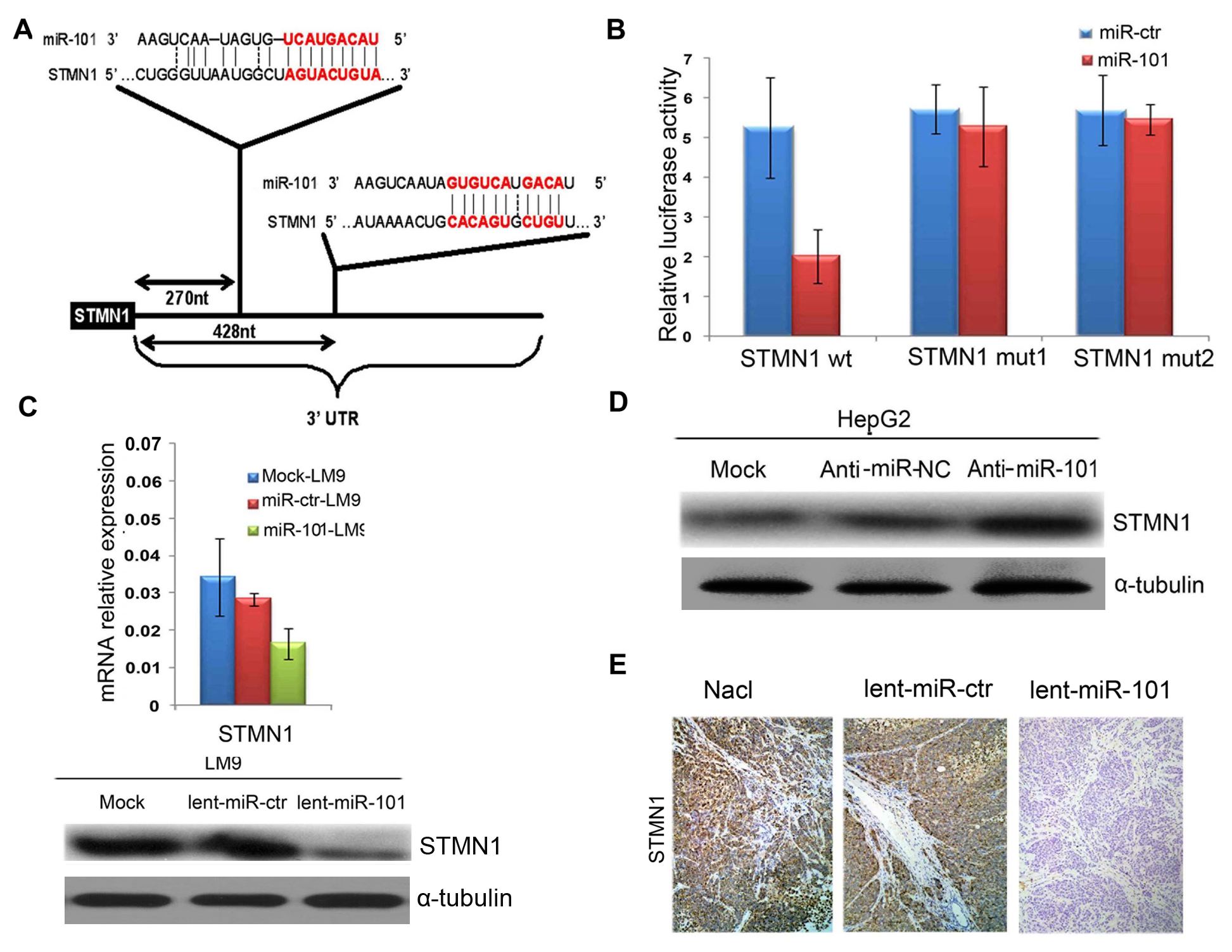

Supplement: S3 Fig — (A) Schematic of predicted miR-101-binding sites in the 3′UTR of STMN1. (B) MiR report constructs containing a wild-type and 2 mutated STMN1 3’UTRs were transfected into LM9 cells, respectively. Relative repression of firefly luciferase expression was standardized to a transfection control. The reporter assays were performed 3 times with essentially identical results. (C) Upper, real-time PCR examination of mRNA levels of ROCK2 between the lenti-miR-101 and control lent-miR-ctr treated LM9 cells. LM9 cells were infected with lent-miR-ctr or lent-miR-101 for 72 hours. Down, ectopic overexpression of miR-101 by lenti-miR-101 reduces the levels of STMN1 protein in LM9 cells, as compared to that in both Mock and lent-miR-ctr treated LM9 cells. (D) Protein expression of STMN1 is up-regulated in HCC HepG2 cells after the down-regulation of miR-101 by anti-miR-101, as compared to that in control Mock and anti-miR-NC HepG2 cells. (E) IHC staining showing down-regulated expressions of STMN1 in HCC tissues of mice treated with systemic delivery of lent-miR-101, as compared to that treated with NaCl or lent-miR-ctr. (TIF) [file pgen.1009960.s001.tif]

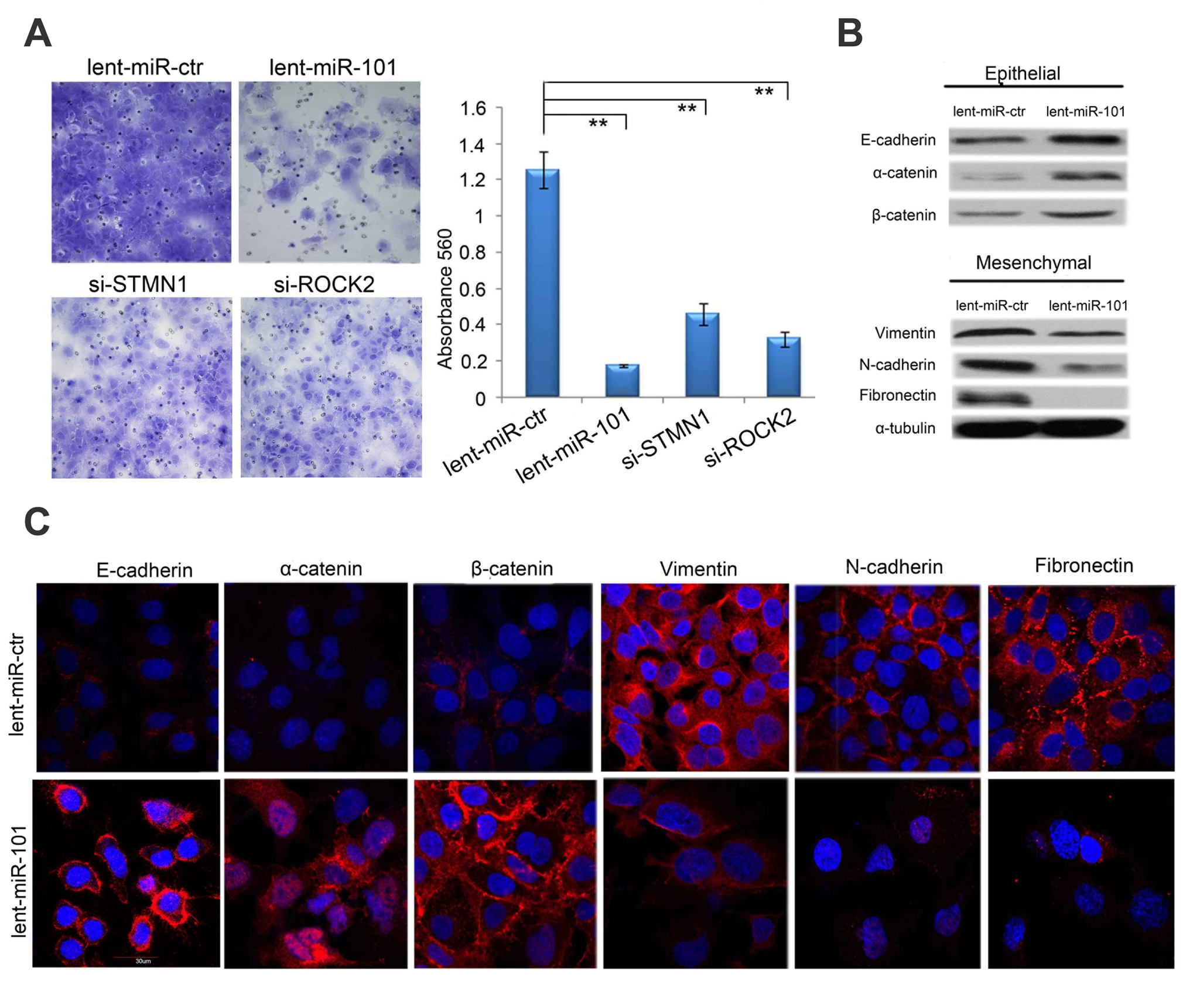

Supplement: S6 Fig — (A) The invasive properties of HCC Huh cells transfected with lent-miR-ctr, lent-miR-101, si-STMN1, and si-ROCK2 were analyzed by an invasion assay using a MatrigelTM Invasion Chamber. Migrated cells were plotted as the average number of cells per field of view from 3 indipendent experiments (**, P<0.01). (B) Expression levels of the epithelial markers E-cadherin, α-catenin, β-catenin and the mesenchymal markers fibronectin, N-cadherin and vimentin were analyzed by Western blot between lent-miR-101 and control lent-miR-ctr treated Huh cells. (C) IF staining was used to compare expression levels/pattern of epithelial markers and mesenchymal markers (red signal) between the control lent-miR-ctr and lent-miR-101 treated Huh cells. The Epithelial markers E-cadherin, α-catenin, β-catenin were upregulated and mesenchymal markers fibronectin, N-cadherin and vimentin were downregulated in lent-miR-101 treated Huh cells, as compare to that in lent-miR-ctr Huh cells. (TIF) [file pgen.1009960.s002.tif]

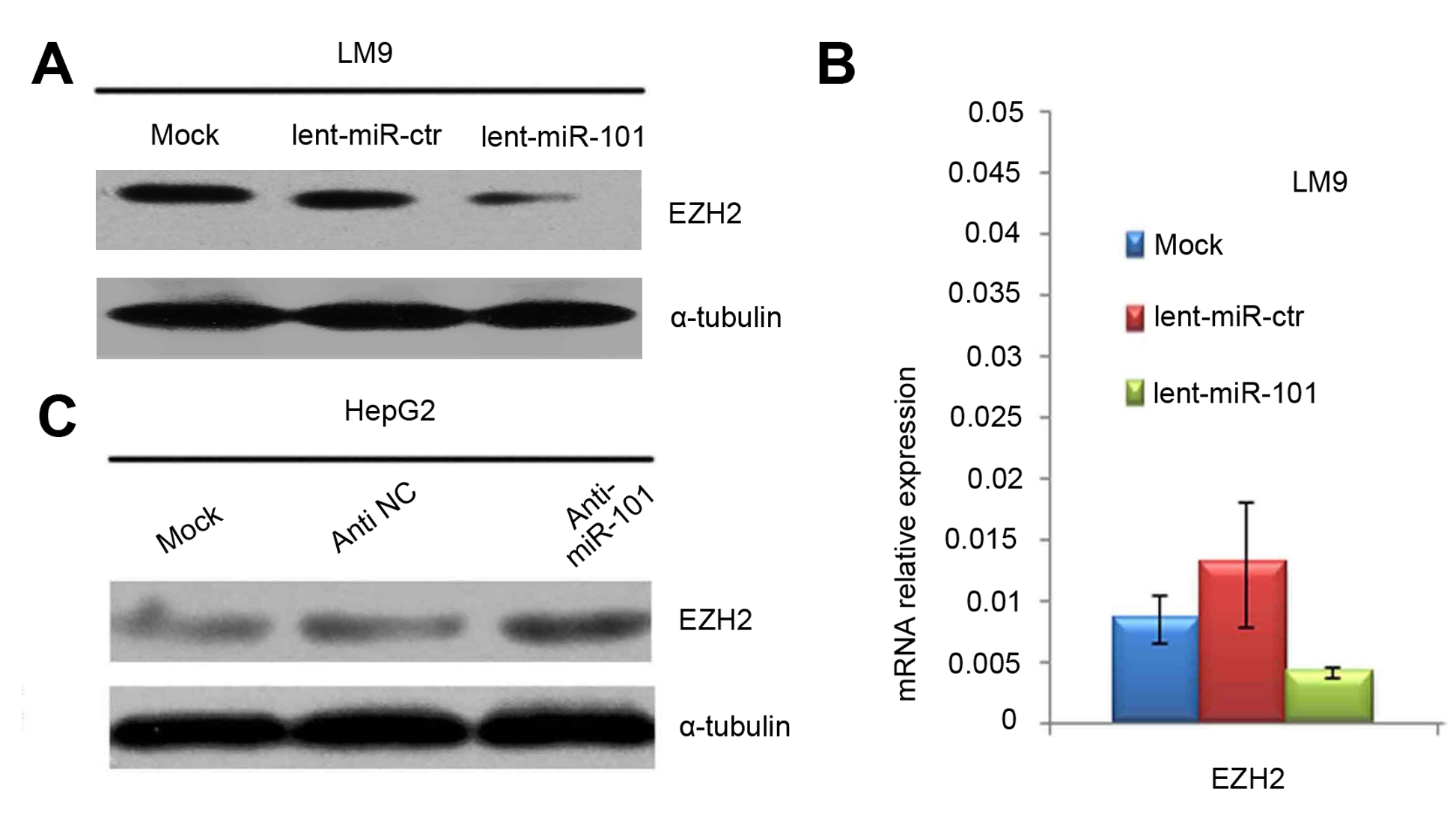

Supplement: S8 Fig — (A) Enforced overexpression of miR-101 in LM9 cells decreases endogenous levels of EZH2 protein. LM9 cells were infected with Mock, lent-miR-ctr or lenti-miR-101 for 72 hours. EZH2 expression was assessed by Western blot. (B) The mRNA levels of EZH2 in Mock, lent-miR-ctr or lenti-miR-101 LM9 cells examined by Real-time PCR. Lenti-miR-101 decreased the levels of EZH2 mRNA in LM9 cells. (C) Western blot assay showing protein levels of EZH2 after the treatment of Mock, Anti-miRNC and anti-miR-101 in HepG2 cell line. Anti-miR-101 could increase EZH2 expression in HepG2 cells. (TIF) [file pgen.1009960.s003.tif]
